# The Human-Specific and Smooth Muscle Cell-Enriched LncRNA SMILR Promotes Proliferation by Regulating Mitotic CENPF mRNA and Drives Cell-Cycle Progression Which Can Be Targeted to Limit Vascular Remodeling

**DOI:** 10.1161/CIRCRESAHA.119.314876

**Published:** 2019-07-23

**Authors:** Amira D. Mahmoud, Margaret D. Ballantyne, Vladislav Miscianinov, Karine Pinel, John Hung, Jessica P. Scanlon, Jean Iyinikkel, Jakub Kaczynski, Adriana S. Tavares, Angela C. Bradshaw, Nicholas L. Mills, David E. Newby, Andrea Caporali, Gwyn W. Gould, Sarah J. George, Igor Ulitsky, Judith C. Sluimer, Julie Rodor, Andrew H. Baker

**Affiliations:** 1 From the Queens Medical Research Institute, BHF Centre for Cardiovascular Sciences, University of Edinburgh, United Kingdom (A.D.M., M.D.B., V.M., K.P., J.H., J.P.S., J.I., J.K., A.S.T., N.L.M., D.E.N., A.C., J.C.S., J.R., A.H.B.); 2 Institute of Cardiovascular and Medical Sciences, BHF Cardiovascular Research Centre, University of Glasgow, United Kingdom (A.C.B.); 3 Institute of Molecular Cell and Systems Biology, College of Medicine, Veterinary and Life Sciences, University of Glasgow, United Kingdom (G.W.G.); 4 School of Clinical Sciences, University of Bristol, Research Floor Level Seven, Bristol Royal Infirmary, United Kingdom (S.J.G.); 5 Department of Biological Regulation, Weizmann Institute of Science, Rehovot, Israel (I.U.); 6 Department of Pathology, Maastricht University Medical Center, the Netherlands (J.C.S., A.H.B.).

**Keywords:** blood vessels, cell cycle, growth factors, interleukins, muscle cells, noncoding RNA, saphenous vein

## Abstract

**Rationale:**

In response to blood vessel wall injury, aberrant proliferation of vascular smooth muscle cells (SMCs) causes pathological remodeling. However, the controlling mechanisms are not completely understood.

**Objective:**

We recently showed that the human long noncoding RNA, *SMILR*, promotes vascular SMCs proliferation by a hitherto unknown mechanism. Here, we assess the therapeutic potential of *SMILR* inhibition and detail the molecular mechanism of action.

**Methods and Results:**

We used deep RNA-sequencing of human saphenous vein SMCs stimulated with IL (interleukin)-1α and PDGF (platelet-derived growth factor)-BB with SMILR knockdown (siRNA) or overexpression (lentivirus), to identify *SMILR*-regulated genes. This revealed a SMILR-dependent network essential for cell cycle progression. In particular, we found using the fluorescent ubiquitination-based cell cycle indicator viral system that SMILR regulates the late mitotic phase of the cell cycle and cytokinesis with *SMILR* knockdown resulting in ≈10% increase in binucleated cells. *SMILR* pulldowns further revealed its potential molecular mechanism, which involves an interaction with the mRNA of the late mitotic protein CENPF (centromere protein F) and the regulatory Staufen1 RNA-binding protein. *SMILR* and this downstream axis were also found to be activated in the human ex vivo vein graft pathological model and in primary human coronary artery SMCs and atherosclerotic plaques obtained at carotid endarterectomy. Finally, to assess the therapeutic potential of *SMILR*, we used a novel siRNA approach in the ex vivo vein graft model (within the 30 minutes clinical time frame that would occur between harvest and implant) to assess the reduction of proliferation by EdU incorporation. SMILR knockdown led to a marked decrease in proliferation from ≈29% in controls to ≈5% with SMILR depletion.

**Conclusions:**

Collectively, we demonstrate that SMILR is a critical mediator of vascular SMC proliferation via direct regulation of mitotic progression. Our data further reveal a potential SMILR-targeting intervention to limit atherogenesis and adverse vascular remodeling.

## Abstract 2

Supplemental Digital Content is available in the text.

**In This Issue, see p 503**

**Meet the First Author, see p 504**

Aberrant proliferation of vascular smooth muscle cells (vSMCs) is a common and functionally important mechanism that impacts on the pathogenesis of many vascular diseases, including intimal thickening associated with remodeling of intravascular stents, coronary artery bypass graft failure, atherosclerosis, and aortic aneurysm formation. In particular, vSMC proliferation is promoted by the injurious microenvironment, partly through the increased exposure of vSMC to inflammatory cytokines and growth factors such as IL (interleukin)-1 and PDGF (platelet-derived growth factor), respectively. These often act in synergy to promote a proliferative phenotype with associated activation of critical gene networks, such as metalloproteinases.^1–4^ Clinically, targeting vSMC proliferation is exceptionally effective at reducing adverse vascular remodeling following balloon angioplasty and vessel stenting, evidenced by extensive research and development of antiproliferative drug-eluting stent technologies.^5,6^ For iatrogenic vascular injury, pathogenic SMC proliferation causes intimal hyperplasia and luminal narrowing of blood vessels in the setting of vascular stenting or vein graft failure.^2,7^ In more complex settings, such as atherosclerosis, vSMC proliferation is central to the accumulation of large numbers of plaque-derived vSMC that not only contribute to the atherogenic process itself but can also confer plaque stabilization.^8,9^ Despite context-dependent heterogeneity in vSMC pathobiology, the underlying activation of vSMC proliferation is a central phenotype to the progression of vessel wall dysfunction. It is, therefore, imperative to further understand the molecular mechanisms that govern vSMC proliferation pathways to advance innovative therapies.

Recently, robust evidence has revealed that noncoding RNAs may play a vital role in the regulation of tissue homeostasis, including cardiovascular homeostasis, and hence pathophysiological conditions.^10^ Mammalian genomes are pervasively transcribed to produce thousands of long noncoding RNAs (lncRNAs). LncRNAs are widely involved in physiological and pathological processes, such as cancer,^11^ autoimmune diseases,^12^ and cardiac disease.^13^ LncRNA can exert their function via a broad range of activities including, but not limited to, chromatin remodeling, formation of nuclear bodies, and activities as scaffolds and guides.^14^ Previous findings have suggested that a substantial proportion of lncRNAs reside within, or are dynamically shuttled to, the cytoplasm to regulate mRNA stability, protein translation, microRNA availability, and impact on protein modifications.^15^ Such RNA-based regulation generally relies on lncRNA interactions with RNA-binding proteins.^16^ We previously identified the lncRNA *SMILR* by RNA-sequencing (RNA-seq) of human vSMCs following activation by IL-1α and PDGF-ββ signaling.^17^ *SMILR* is an intergenic and poorly conserved lncRNA that consists of only a single 3-exon polyadenylated transcript that is vSMC-enriched, and its knockdown by RNA interference blocked IL-1α and PDGF-ββ-induced (IL1-PDGF) vSMC proliferation.^17^ Thus, we reasoned that identifying the downstream targets and binding partners of *SMILR* would reveal the specific mechanism by which it regulates vSMC proliferation and hence provide a novel therapeutic target for preventing adverse vascular remodeling.

## Methods

The authors declare that all supporting data and materials are available within the article (and its in the Online Data Supplement) and available from the corresponding author on reasonable request. All RNA-seq data have been made deposited in the Gene Expression Omnibus repository, study number GSE120521 for the atherosclerosis RNA-seq and GSE117608 for SMILR RNA-seq.

Expanded information about materials and methods are available in the Online Data Supplement.

### Declaration of Helsinki

All studies comply with the Declaration of Helsinki, that the locally appointed ethics committee has approved the research protocol and that informed consent has been obtained from the subjects.

Primary human saphenous vein SMCs (HSVSMCs) were isolated via explant technique from consented patients and cultured as previously described.^17^ All procedures had local ethical approval (15/ES/0094). HSVSMCs from passage 3 to 5 were used for this study, and cells were synchronized in DMEM containing 0.2% FBS for 48 hours before experimentation. Modulation of *SMILR* expression was performed through the utilization of dsiRNA or SMILR lentivirus with appropriate controls and their effect on the genome was assessed via RNA-seq and confirmed through subsequent quantitative real-time polymerase chain reaction (qRT-PCR) validation. All qRT-PCR data were analyzed via the 2-ΔΔCt method.^18^ This method uses a house keeping gene and UBC (ubiquitin C) was selected as a housekeeping gene due to its stability across all groups and conditions studied. Data are graphed as relative quantification normalized to the UBC housekeeping gene.^18^

Assessment of siRNA-SMILR on SMC cell cycle was performed via fluorescence ubiquitin cell cycle indicator (FUCCI)-fluorescence-activated cell sorter (FACS) analysis and confocal imaging for the percent of binucleated cells. SiRNA targeting *AURKB* was used as a positive control in these studies as previously described.^19,20^

To evaluate binding partners of *SMILR*, antisense SMILR, or GFP (green fluorescent protein) probes were designed. For each pulldown experiment, either 5 GFP or 5 SMILR probes were used to capture bound RNA. Before RNA extraction, RNA was spiked with 75 ng of total *Caenorhabditis* *elegans* RNA and AMA1 used as a reference gene as previously described.^21^ qRT-PCR was used to assess RNA expression.

To assess if SMILR exhibited any venous/arterial differences in expression or function, human coronary artery SMCs (HCASMC) were use and cultured under the same conditions as HSVSMCs. Stimulation of these cells was performed under basal and IL-1/PDGF-BB stimulated conditions as described in Ballantyne et al^17^ and assessment of the effect of siRNA-SMILR on HCASMC proliferation (Edu-FACS), binucleation (confocal imaging), and downstream target expression (qRT-PCR) was performed.

To address if SMILR exhibited any protein binding partners, SMILR protein pulldowns were performed using streptavidin magnetic beads to capture the biotinylated RNA target and any bound proteins from stimulated SMCs. Mass spectrometric analysis was used to identify proteins for subsequent downstream analysis and validation. Anti-Stau1 (Staufen 1) pulldowns were used as validation with appropriate IgG control to confirm SMILR and other RNA target binding by qRT-PCR.

Similar to Ballantyne et al^17^ patients with symptomatic carotid artery stenosis scheduled to undergo carotid endarterectomy were recruited from neurovascular clinics at the Royal Infirmary of Edinburgh to undergo separate [18F]-fluoride and [18F]- fluorodeoxyglucose positron emission tomography combined with computed tomography scans. Regions of stable and unstable plaque were denoted by low and high tracer uptake respectively and appropriately dissected. RNA-seq was performed to assess transcriptomic differences between plaque sections and SMILR expression assessed via qRT-PCR. In situ hybridization was used to visualize the localization of SMILR within the plaque regions.

To assess the potential clinical utilization of siSMILR, segments of human saphenous vein obtained from consented patients undergoing bypass surgery were pinned down with minutien pins on a Sylgard coated dissection dish with the luminal surface facing upward for 0, 7, or 14 days with media refreshed every 2 days. At day 0 and after 7 and 14 days of culture, the vein segments were washed in PBS and snap-frozen for subsequent RNA extraction or fixed in 4% PFA for histology. Proliferation of segments was assessed through the utilization of Click-iT EdU Alexa Fluor 488 In Vivo Imaging Kit. Expression levels of SMILR and target RNA were assessed through qRT-PCR analysis. For siRNA transfections, HSV segments cut in equal pieces of ≈1 cm^2^ were bathed in PBS containing 25 μmol/L siSMILR and scrambled siRNA control for 30 minutes in 24-well plate. After 30 minutes of incubation, the vein segments were washed with PBS and pinned down as described above. To confirm siRNA transfection, cy3-tagged SMILR was transfected and visualized along with DAPI (4′,6-diamidino-2-phenylindole) and α-smooth muscle actin costaining via confocal imaging.

Samples of ≥n=5 were subjected to the Shapiro-Wilk test to assess normal distribution followed by Student *t* test or ANOVA. Normal distribution cannot be determined on small samples sizes and samples with n<5 were assumed to be not normally distributed and subjected to Iman and Conover nonparametric ranking followed by Student *t* test or ANOVA. Statistical significance *P*<0.05 under all conditions.

## Results

### Manipulation of SMILR Expression Identifies a Target Cell Cycle Network in HSVSMCs

**Figure 1.**
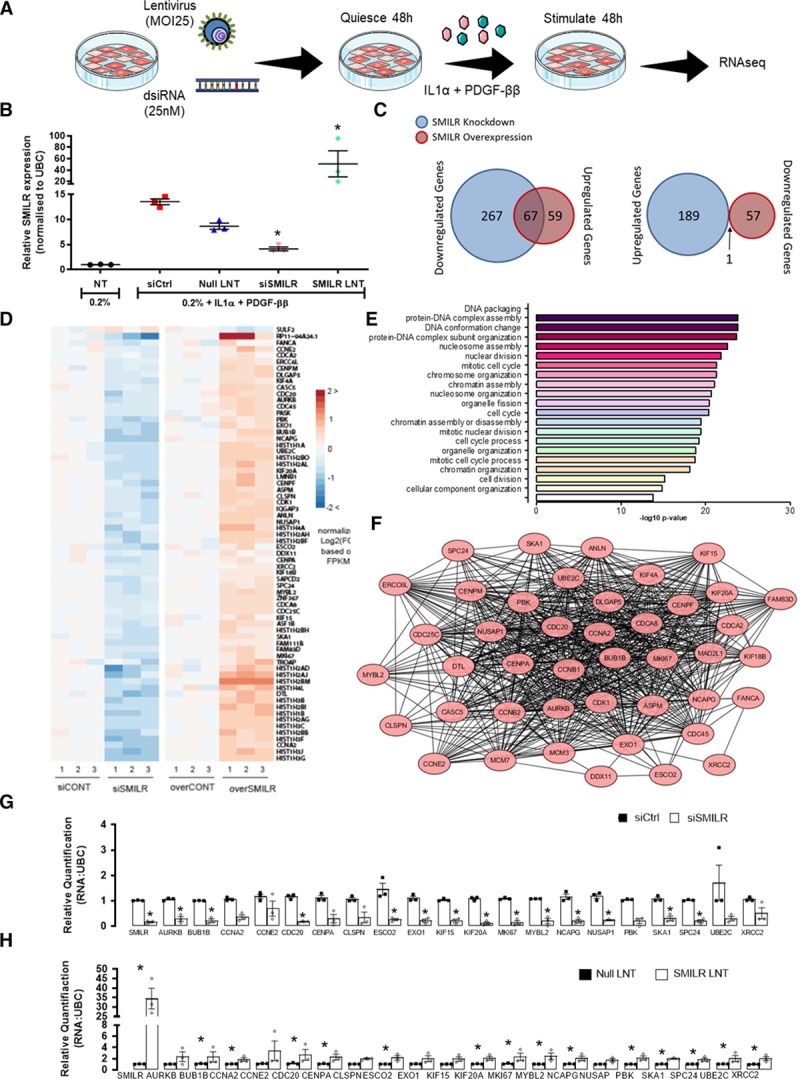
**Transcriptomics identifies a cell cycle-associated network targeted by SMILR in proliferative vascular smooth muscle cells (vSMCs**). **A**, Schematic of experimental design for *SMILR* knockdown and overexpression using dsiRNA and lentivirus (LNT), respectively. **B**, Validation of *SMILR* knockdown and overexpression via qRT-PCR (quantitative real-time polymerase chain reaction) of 3 technical replicates used for RNA-sequencing (RNA-seq) from one patient sample. **C**, Venn diagrams illustrating the number of genes dysregulated by *SMILR* knockdown and overexpression. **D**, Heatmap of all overlapping 68 significant changes observed on *SMILR* depletion and overexpression. Fold change calculated compared to the average FPKM (fragments per kilobase of transcript per million mapped reads) in the control (CONT) samples. **E**, Gene Ontology (GO) terms for the *SMILR*-regulated gene cohort. **F**, Protein network of 40 proliferative and cell cycle-associated genes found to be dysregulated with *SMILR* depletion and overexpression. (*Continued* )**Figure 1 Continued. G** and **H**, Further validation by qRT-PCR of the top 20 identified dysregulated proliferative and cell cycle-associated genes with siRNA control (siCtrl) vs siSMILR and control LNT (null LNT) vs SMILR LNT. IL indicates interleukin; MOI, multiplicity of infection; and PDGF, platelet-derived growth factor. *P<0.05, by Iman and Conover nonparametric ranked paired Student *t* test of deltaCT values between gene of interest and the housekeeper gene UBC (ubiquitin C), n=3 biological replicates.

*SMILR* depletion and overexpression were previously shown to decrease and increase, respectively, proliferation induced by stimulation of HSVSMCs with IL1-PDGF.^17^ However, no characterization of the mechanisms of regulation of *SMILR* by IL1-PDGF was described. Accordingly, we sought to identify the potential transcription factor binding sites within the promoter region of *SMILR* (Online Figure IA). Within the 2000 bp upstream of *SMILR’s* transcription start site, we identified binding sites for transcription factors that are activated by IL-1α and PDGF-BB, including NF-KB (nuclear factor kappa-light-chain-enhancer of activated B cells), CEBP-β (CCAAT enhancer-binding protein beta), ETS1 (ETS proto-oncogene 1), AP1 (activator protein 1), NFAT (nuclear factor of activated T cells), IRF8 (interferon regulatory factor 8), MYB (MYB proto-oncogene), and AML (acute myeloid leukemia 1; Online Figure IB). Of the commercially available transcription factor inhibitors, we analyzed the subsequent effects on *SMILR* expression following IL1-PDGF stimulation. This identified that the upregulation of *SMILR* following IL1-PDGF stimulation may, in part, be because of activation of NF-KB (Online Figure IC and controls in Online Figure ID). Interestingly, SMILR overexpression does not trigger quiesced HSVSMCs to proliferate in the absence of IL1-PDGF stimulation (Online Figure II). Therefore, to further determine the downstream effects of manipulation of *SMILR* levels on proliferation, RNA-seq was performed on stimulated HSVSMCs exposed to either *SMILR* depletion via siRNA (siSMILR) or overexpression via lentivirus treatment to identify a downstream SMILR-dependent transcriptome (Figure 1A).

Quantification of miR-146a, -221 and -222 by qRT-PCR confirmed activation of the IL-1α and PDGF-ββ signaling pathways, respectively (Online Figure III).^22,23^ Alterations in *SMILR* expression levels were validated by qPCR (Figure 1B). Considering a fold change ≥1.5 and an adjusted false discovery rate *P*<0.05, 523 (334 downregulated and 189 upregulated) and 183 (126 upregulated and 57 downregulated) transcripts were significantly differentially expressed following knockdown or overexpression, respectively (Figure 1C). As we observe opposing effects on proliferation with *SMILR* knockdown and overexpression, we focused on the transcripts that were dysregulated in opposing levels. This revealed 68 transcripts (Figure 1C and 1D) indicating that such an approach might be powerful in identifying a distinct *SMILR*-targeted biological interactome. This set of *SMILR*-regulated genes was enriched for cell division-related and nucleosome assembly gene ontology terms (Figure 1E). Interestingly, analysis by STRING (Search Tool for the Retrieval of Interacting Genes/Proteins)^24^ identified that 59% (n=40 of 68 genes) of the overlapping genes were associated with a single network involved in progression through the cell cycle, primarily the mitotic phase (Figure 1F). The top 20 genes identified by RNA-seq that were differentially regulated genes by *SMILR* (Online Figure IV) were selected for further validation in 3 different patient saphenous vein derived SMCs by qRT-PCR. We observed consistent and robust opposing regulation of the network following *SMILR* depletion and overexpression in HSVSMCs stimulated with IL1-PDGF (Figure 1G and 1H). In agreement with the absence of proliferation phenotype after *SMILR* overexpression in nonstimulated quiescent conditions, *SMILR* overexpression in nonstimulated quiescent HSVSMCs did not result in transcription changes of the identified network (Online Figure IIB). Collectively, these data suggest that *SMILR* mechanistically targets the vSMC cell cycle in response to IL1-PDGF stimulation.

### Manipulation of SMILR Expression Effects Cell Cycle Progression in vSMC

**Figure 2.**
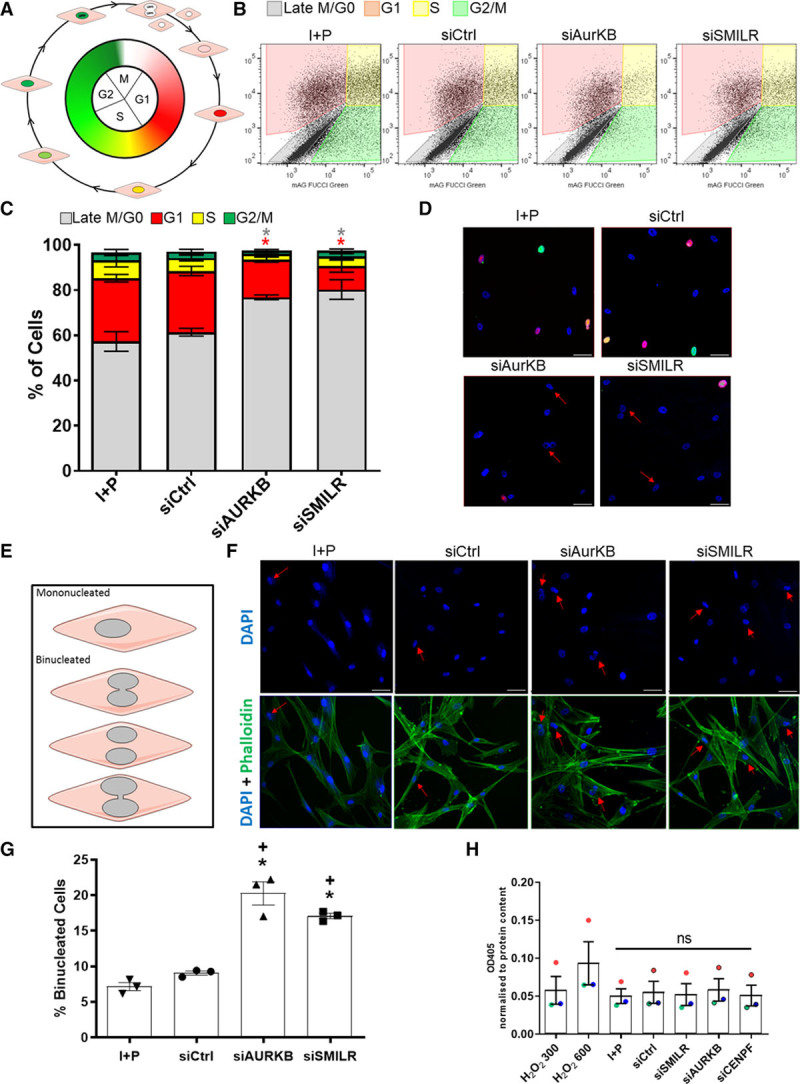
**SMILR manipulation regulates the cell cycle of vascular smooth muscle cells (vSMC**). **A**, Schematic of fluorescence ubiquitin cell cycle indicator (FUCCI) viral analysis. **B**, Flow cytometric analysis tracking cell cycle changes indicated by color changes of the FUCCI viruses. **C**, Bar chart representing average changes in the % of cells in each stage of the cell cycle. Repeated measures ANOVA for *P<0.05, Iman and Conover ranked nonparametric analysis followed by 1-way ANOVA, n=3 biological replicates. **D**, Fluorescent microscopy of humans saphenous vein derived smooth muscle cells (HSVSMCs) infected with the FUCCI viral system. Scale bars at 50 µm. Red arrows indicate binucleated cells. **E**, Schematic of characterization method of binucleated cells. **F**, Fluorescent images of HSVSMCs stained with DAPI (4′,6-diamidino-2-phenylindole) and phalloidin. Scale bars at 50 µm. Red arrows indicate binucleated cells. (*Continued* )**Figure 2 Continued. G**, Bar chart representing the % of total cells that were binucleated. *P<0.05, Iman and Conover ranked nonparametric analysis followed by 1-way ANOVA vs IL (interleukin)-1α/PDGF (platelet-derived growth factor)-ββ treatment alone (I+P); + = *P*<0.05 vs siControls, n=3 biological replicates. **H**, Bar chart representing caspase-3 activity in HSVSMCs cultured with siRNA or hydrogen peroxide as a positive control. Caspase activity measured by OD405. n=3 biological replicates, vs I+P treatment. ns indicates not significant.

Next, we functionally assessed *SMILR’s* ability to directly target cell cycle progression in HSVSMCs. First, we used the FUCCI viral system as well as flow cytometric analysis to track the cell cycle in synchronized HSVSMCs stimulated with IL1-PDGF for 96 hours with and without *SMILR* knockdown. After FUCCI viral infection, cells in G0/G1 and S/G2/early M cell cycle phases express mCherry and mAzami-Green, respectively.^25^ Figure 2A represents the color change predicted in cycling cells, dependent on the relative stage of cell cycle—red (G1), yellow/orange (G1/S), green (G2/early M), or colorless (late M/G0) fluorescence. AurKB (aurora kinase B), a well-known cell cycle and mitotic mediator, was used as a positive control. Notably, it is also one of *SMILR’s* downstream targets in the interactome (Figure 1F). Consistent with previous findings showing IL1-PDGF stimulation only promotes 30% of quiescent cells to proliferate,^17^ ≈60% of the FUCCI-infected HSVSMCs stimulated with IL1-PDGF were found to be colorless under control conditions (Figure 2B and 2C). Effective knockdown of *AurKB* in the HSVSMCs (4±0.48-fold reduction compared to control; Online Figure V) resulted in a cell cycle defect with a decrease in the G1 phase (*P*<0.05) and concurrent increase in M/G0 phase (*P*<0.05; Figure 2B and 2C). Analysis on FUCCI-infected cells also revealed a clear defect in the G1 phase and increase in the late M/G0 phase of the cell cycle following treatment with siSMILR, thereby phenocopying the effect of *AurKB* knockdown (Figure 2C). A hallmark of such a mitotic phase defect is the inability to correctly segregate daughter from mother cells during cytokinesis, resulting in cellular binucleation,^26^ which was evident in both FUCCI-infected cells treated with siAurKB and siSMILR (Figure 2D). Accordingly, cells treated with siSMILR and siAURKB were stained with DAPI and phalloidin and assessed for binucleation via fluorescent microscopy (Figure 2E and 2F). This revealed an increase in the percentage of binucleated cells from 7.2±0.6% and 9.1±0.3% in nontransfected and siControls, respectively, to 17.1±0.4% following *SMILR* depletion (*P*<0.05), and a similar phenotype was observed in siAurKB-treated cells (20.3±1.6% binucleation; Figure 2G). Importantly, siRNA treatment had no significant effect on apoptosis with any of the siRNA-based treatments (Figure 2H). Taken together, these data implicate a function for *SMILR* in regulating the late mitotic phase of cell cycle in vSMCs.

### SMILR Directly Targets CENPF in the Cell Cycle Network

**Figure 3.**
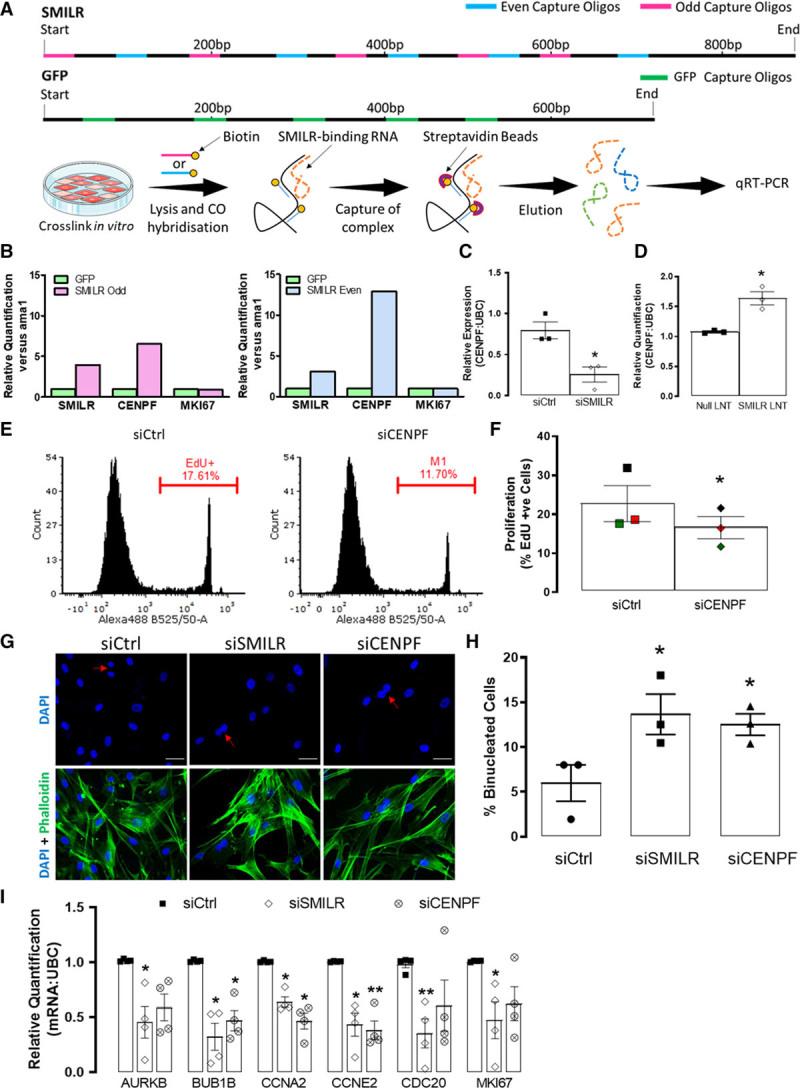
**RNA: RNA analysis reveal a SMILR: CENPF (centromere protein F) interaction.** **A**, Schematic of DNA antisense biotinylated probes site for *SMILR* and *GFP* and the experimental design of RNA: RNA pulldowns. **B**, Bar charts representing relative enrichments of *SMILR*, *CENPF*, and MKI67 (marker of proliferation Ki-67) in SMILR-even and -odd pulldowns vs the GFP (green fluorescent protein) control pulldown. Each even and odd SMILR probe pulldown was performed once across 2 independent biological replicates. Effects of (**C**) *SMILR* knockdown and (**D**) *SMILR* overexpression on *CENPF* mRNA. **P<0.05 Iman and Conover nonparametric ranked analysis followed by Student *t* test, n=3 biological replicates. **E**, Representative fluorescence-activated cell sorter (FACS) histogram plots depicting EdU uptake in siCtrl and siCENPF treated humans saphenous vein derived smooth muscle cells (HSVSMCs). Gate represents EdU+ cells. **F**, Bar chart showing mean changes of EdU incorporation in siCtrl and siCENPF treated HSVSMCs. (*Continued* )**Figure 3 Continued.** Iman and Conover ranked nonparametric analysis followed by *t* test, n=3 biological replicates. *P<0.05. **G**, Fluorescent images of HSVSMCs stained with DAPI (4′,6-diamidino-2-phenylindole) and phalloidin. Scale bar represents 50 µm. Red arrows indicate binucleated cells. **H**, Bar chart representing the % of total cells that were binucleated. Iman and Conover ranked nonparametric analysis followed by Student *t* test *P<0.05 vs siCtrl, n=3 biological replicates. **I**, The effects of *CENPF* knockdown on mitotic associated genes compared to effects observed with knockdown of *SMILR*, *P<0.05, by Iman and Conover ranked nonparametric analysis followed by paired *t* test, n=4 biological replicates.

With both overexpression^17^ and knockdown (Online Figure IV) approaches affecting *SMILR* expression levels predominantly in the cytoplasmic fraction, we, therefore, reasoned that *SMILR* could directly regulate the identified affected genes by binding to the mRNA in the cytoplasm. We used a database of predicted lncRNA-RNA interactions by Terai et al^27^ and considered the top 100 genes predicted to interact with *SMILR* based on SumEnergy (Online Table I). These genes were analyzed in terms of expression level in stimulated vSMC, differential expression in *SMILR* depleted or *SMILR* overexpressed conditions, as well as differential expression on stimulation with IL1-PDGF (see filtering details in Online Data Supplement methods and summary in Online Table I). This revealed that *CENPF*, a mitotic centromere protein, was the highest-ranked mRNA predicted to interact with *SMILR* (minimum and sum energy of −35 and −2631 kcal/mol, respectively, Online Table I). The predicted interacting base pair region of the *SMILR*/*CENPF* mRNA interaction extends across 51 base pairs (39–90) within the sequence of *SMILR* and 58 base pairs (3291–3349) within the coding sequence of *CENPF* transcript (Online Figure VII). We used RNA antisense pulldown followed by qRT-PCR to confirm this predicted interaction. Two sets (5 even and 5 odd) of 3′-biotinylated DNA capture oligonucleotides were designed to hybridize specifically to *SMILR* (Online Data Supplement).^28,29^ One set of 5 GFP-specific 3′-biotinylated DNA capture oligonucleotides were also used as a negative control. A schematic overview of the experimental design is provided in Figure 3A. The relative enrichment of *SMILR* and the *CENPF* mRNA present in both the *SMILR*-even and -odd pools was calculated with respect to the GFP pool, which was used as background reference. We observed a 3- and 4-fold enrichment of *SMILR* with the even and odd probes, respectively. *CENPF* transcript was also coenriched by 13- and 7-fold in the even and odd *SMILR* pulldowns, respectively, thereby independently validating the predicted interaction between *SMILR* and *CENPF* mRNA. Importantly, MKI67 (marker of proliferation Ki-67) mRNA another downstream target within the *SMILR*-dependent cell cycle network was assessed in the even and odd *SMILR* pulldowns and found to not to be enriched—suggesting specificity for a *SMILR*: *CENPF* mRNA interaction within the interactome (Figure 3B). Additionally, in agreement with the RNA-seq data, *SMILR* depletion and overexpression led to a downregulation and upregulation of *CENPF* transcript levels, respectively (Figure 3C and 3D). While having no effect on apoptosis, *CENPF* depletion (Online Figure VIII) resulted in a significant decrease in EdU incorporation (Figure 3E and 3F). Additionally, similar to previous findings,^30,31^ *CENPF* depletion resulted in an increase in the percentage of binucleated cells (12.5±1.2%) comparable to *SMILR* knockdown (13.7±2.2%), which was significantly greater than that observed under control conditions (6.0±2.0%, *P*<0.05, Figure 3G and 3H). Importantly, knockdown of *CENPF* also phenotypically mimics the effects of *SMILR* knockdown on key genes within the cell cycle network (Figure 3I). Thus, these data support the concept that *SMILR* positively targets *CENPF* mRNA, which is critical for vSMC proliferation.

To further examine the temporal involvement of *SMILR* and *CENPF* transcripts in promoting IL1-PDGF induced proliferation, time-course experiments were used and show that *SMILR* expression is significantly upregulated before significant EdU+ incorporation and *CENPF* mRNA expression is detected (Online Figure IX). Taken together, this suggests that *SMILR* is required at the early stages of IL1-PDGF stimulation to promote the induction of proliferation and mitotic progression.

### The SMILR:CENPF RNA Interaction Is Regulated by Staufen1

**Figure 4.**
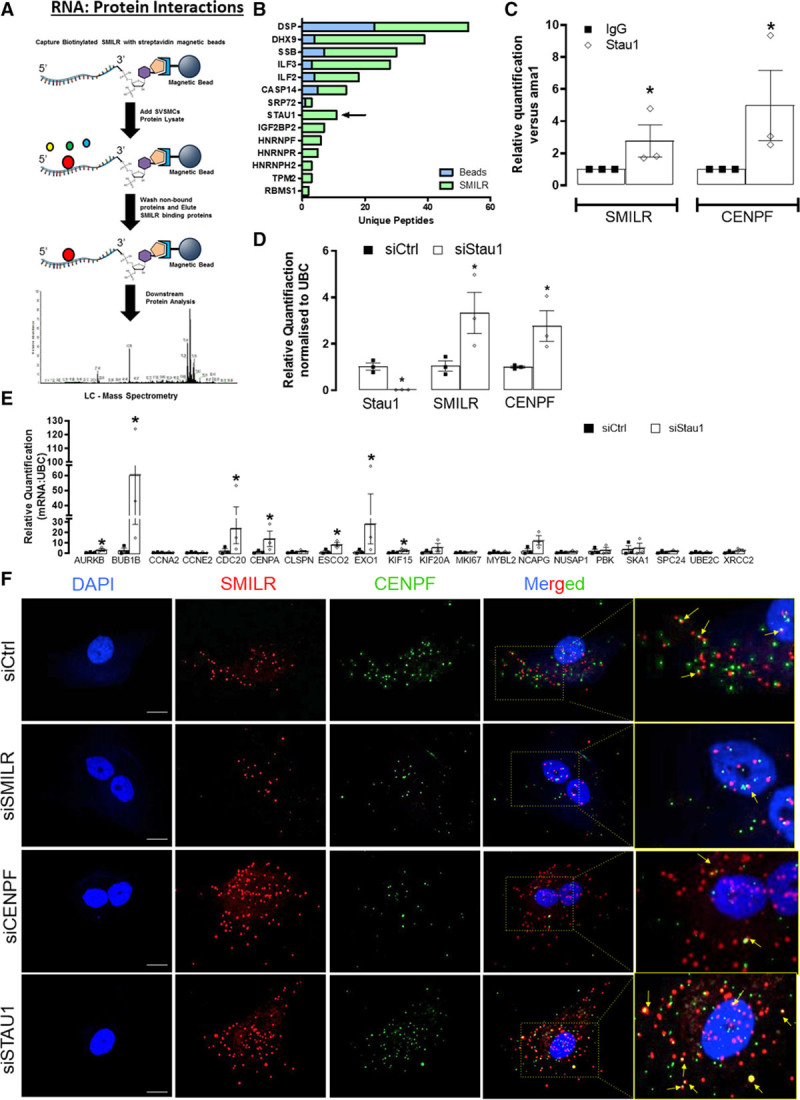
**STAU1 (Staufen 1) degrades the SMILR: CENPF (centromere protein F) interaction to mediate vascular smooth muscle cell (vSMC) proliferation.** **A**, Schematic showing methodology of biotinylated *SMILR* pulldowns. **B**, Mass spectrometry identified 14-enriched *SMILR*-binding proteins. STAU1 was specifically enriched in the *SMILR* pulldown with 11-unique peptides detected. **C**, Anti-STAU1 pulldowns confirming interaction with *SMILR* and *CENPF*; *P<0.05, by Iman and Conover ranked nonparametric analysis followed by *t* test, n=3 pulldowns from 3 independent patient samples. **D**, Relative quantification of STAU1, *SMILR*, and *CENPF* expression with SiSTAU1 at 10 nmol/L. *P<0.05, by Iman and Conover ranked nonparametric analysis followed by *t* test, n=3 biological replicates. **E**, The effects of STAU1 knockdown on the top 20 downregulated cell cycle-associated genes regulated by *SMILR*, *P<0.05, by Iman and Conover ranked nonparametric analysis followed by *t* test, n=3 biological replicates. **F**, RNA fluorescence in situ hybridization (FISH) for *SMILR* (red) and *CENPF* (green) in stimulated vSMCs under control, siSMILR, siCENPF, and siSTAU1 conditions. Scale bar represents 20 µm. Yellow arrows show some colocalization events.

RNAs, including lncRNAs, have been found to occasionally contain structural motifs that can interact with other RNAs to form functional RNA-RNA hybrids, which can then recruit proteins that regulate their function or stability.^32^ Accordingly, to understand whether the function of *SMILR/CENPF* RNA hybrid is dependent on an RNA:protein binding interaction, we performed pulldowns using 3′-desthiobiotin-labeled full-length *SMILR* and protein lysates from IL1-PDGF stimulated HSVSMCs (Figure 4A). Mass spectrometry identified 14 potential *SMILR*-binding proteins (Figure 4B). The STAU1, known to be involved in mRNA decay and binds lncRNA and mRNA hybrids,^33^ was clearly enriched in *SMILR* pulldowns when compared with beads alone or control 3′-desthiobiotin-labeled full-length GFP pulldowns (Online Figure X). Moreover, previous reports have suggested that STAU1 is involved in checkpoint decisions in G2 and/or G2/M transitions, which intersects with the cell cycle defects observed with siSMILR.^34^ Hence, STAU1 seems to be a prime candidate partner for SMILR’s mechanism of action. Immunoprecipitation of STAU1 from HSVSMC lysates stimulated with IL-1 PDGF followed by qRT-PCR revealed enrichment of *SMILR* by 2.8±1-fold (*P*<0.05) when compared with IgG controls (Figure 4C), validating the mass spectrometry results. Additionally, we found that STAU1 is likely to bind to *SMILR* within the first half of its sequence, which as mentioned above, is the predicted interaction site with CENPF (Online Figures VII and XI). We also identified coenrichment of *CENPF* in the STAU1 pulldowns by 5.0±2.2-fold (*P*<0.05; Figure 4C). To explore the involvement of STAU1 in controlling the proliferative phenotype mediated by *SMILR* and *CENPF*, we knocked down STAU1 using dsiRNA and revealed an increase of *SMILR* and *CENPF* mRNA by 3.3±0.9- and 3.0±1.1-fold, respectively (Figure 4D). We accordingly sought to further examine the effect of STAU1 knockdown on the *SMILR* downstream targets. Analysis of the same 20 targets described in Figure 1, which are downregulated and upregulated following SMILR knockdown and overexpression, revealed that 7 of these genes were significantly upregulated with STAU1 knockdown (Figure 4E). Using RNA fluorescence in situ hybridization, we were also able to examine the colocalization of *SMILR* and *CENPF* with STAU1 knockdown (Figure 4F). This revealed that, when compared with control conditions, SMILR/CENPF transcript colocalization is not dependent on STAU1 expression and that there seems to be increased SMILR/CENPF colocalization events with STAU1 KD (Figure 4F). Collectively, these data suggest that once *SMILR* expression is upregulated in IL1-PDGF conditions, it is able to bind to *CENPF* mRNA. This may subsequently counteract STAU1-mediated regulation, thereby culminating in a proliferative environment and cell cycle progression in vSMCs.

### SMILR and the Targeted Cell Cycle Network Are Activated in Atherosclerosis and Ex Vivo Vein Model of Human Saphenous Vein

**Figure 5.**
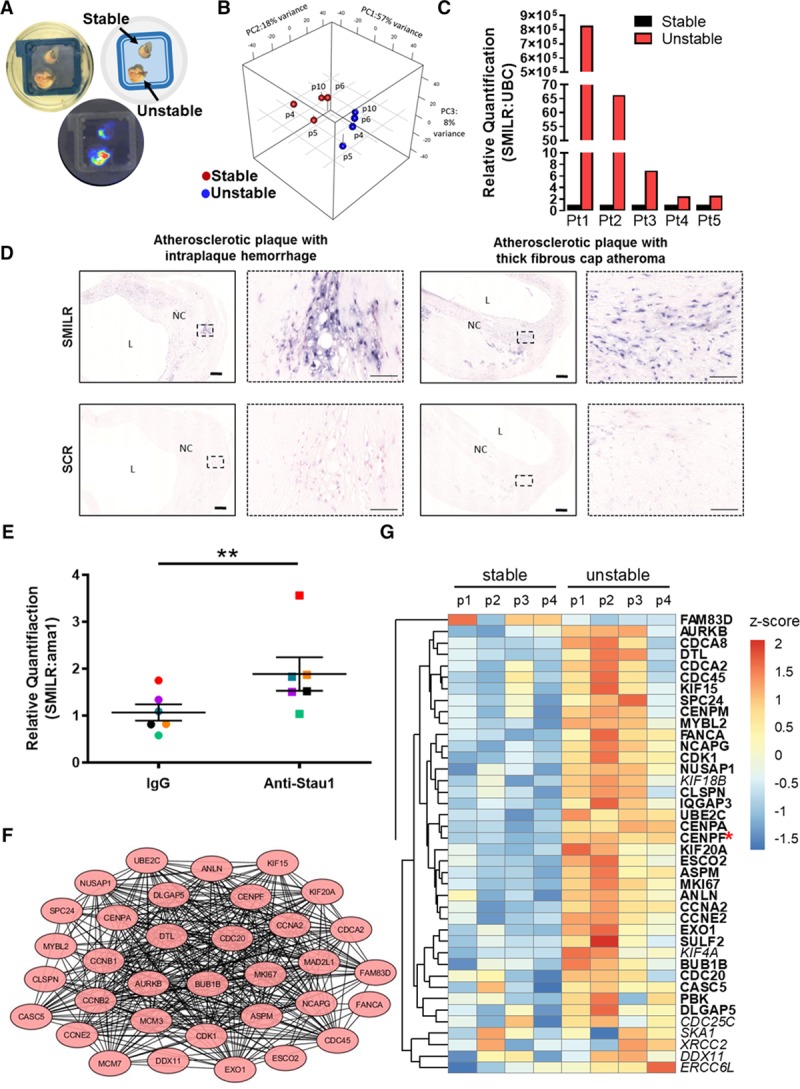
**SMILR and the targeted cell cycle network are activated in atherosclerosis.** **A**, Ex vivo 18F imaging of unstable vs stable plaques. **B**, Principal component analysis (PCA) of the RNA-sequencing (RNA-seq) of the stable and unstable samples. **C**, Relative FPKMs (fragments per kilobase of transcript per million mapped reads) of protein-coding genes linked with plaque instability, including those associated with inflammation and calcification. **D**, Representative images of in situ detection of *SMILR* in plaques obtained from the carotid artery derived from symptomatic patients at carotid endarterectomy (n=5 biological replicates per plaque type, replicates in Online Figure XII). *SMILR* is visualized using NBT/BCIP (nitro-blue tetrazolium and 5-bromo-4-chloro-3'-indolyphosphate; purple) at varying intensities across plaques exhibiting either intraplaque hemorrhage or thick fibrous cap. Nuclei are stained with fast red. Scale bar represents 200 μm. **E**, *SMILR* enrichment in STAU1 (Staufen 1) pulldowns in whole carotid plaques, n=6, **P<0.01, by paired Student *t* test with paired experiments matched by color. Protein network (**F**) and heatmap (**G**) of the 34 proliferative and cell cycle-associated genes found to be dysregulated with *SMILR* manipulation and unregulated in unstable plaques. L indicates arterial lumen; and NC, lipid core.

Despite context-dependent heterogeneity in vSMC pathobiology, defects in SMC cell cycle and hence proliferation, are hallmarks of vascular pathologies including atherosclerosis and neointimal hyperplasia associated with vein graft disease.^3,4,9^ As *SMILR* is poorly conserved, we are limited to human disease and not animal models to study disease association and causality. To interrogate the SMILR:CENPF:STAU1 interaction in human atherosclerosis, we performed an RNA-seq on relatively stable and unstable regions dissected from fresh human carotid plaques obtained at carotid endarterectomy in symptomatic patients. Although classified as stable, these plaques may still contain regions of instability. This is demonstrated by ex vivo 18F-sodium fluoride imaging of explanted plaques, which was used to confirm the appropriate segregation by regions of relatively more unstable versus stable plaque, where increased uptake of the radiotracer^35^ was more apparent in unstable dissections and less so in the stable regions (Figure 5A). Importantly, Principal Component Analysis of the RNA-seq showed a clear clustering of the distinct regions separately and not clustering together within each patient sample (Figure 5B). The differential expression analysis confirmed the changes of protein-coding genes linked with plaque instability, including those associated with inflammation, matrix remodeling, and calcification (Online Figure XII). *SMILR* expression was upregulated in all unstable plaque samples assessed by qRT-PCR (Figure 5C). Additionally, SMILR was detected using in situ hybridization with varying intensity across all carotid atherosclerotic plaques from symptomatic patients (Figure 5D, Online Figure XIII). STAU1 pulldowns in whole carotid plaques also further revealed an interaction with *SMILR* with a 2-fold enrichment compared with IgG controls (*P*<0.01; Figure 5E). Remarkably, we also observed that 32 of the 40 *SMILR*-dependent cell cycle interactome were also upregulated within the unstable plaques compared with stable, including *CENPF* (Figure 5F and 5G). Collectively, these data suggest that the *SMILR*/*CENPF*-STAU1 axis is activated in unstable atherosclerosis.

**Figure 6.**
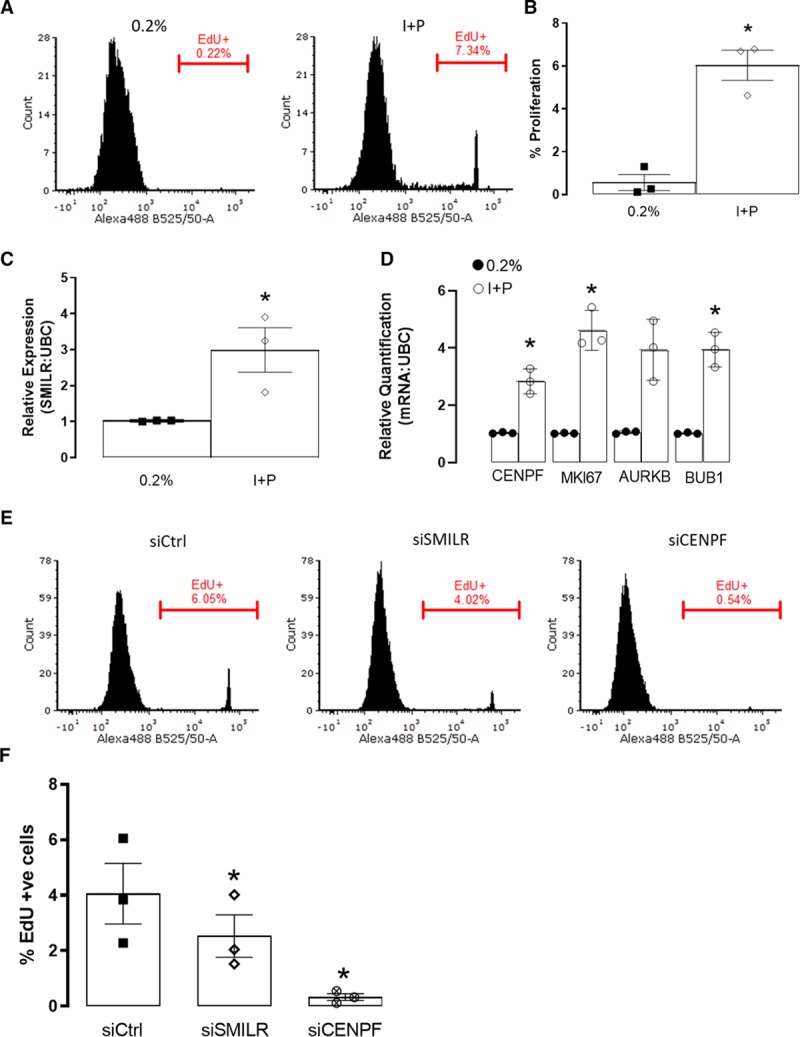
**Role of SMILR in human coronary artery smooth muscle cells (HCASMCs**). **A**, Representative fluorescence-activated cell sorter (FACS) histogram plots depicting EdU uptake in 0.2% and I+P treated HCASMCs. Gate represents EdU+ cells. **B**, Bar chart showing mean changes of EdU incorporation in 0.2% and I+P conditions. Iman and Conover ranked nonparametric analysis followed by *t* test, n=3 biological replicates. *P<0.05. Bar charts showing relative expression of (**C**) SMILR and (**D**) CENPF (centromere protein F), MKI67 (marker of proliferation Ki-67), AURKB, and CDC20 by quantitative real-time polymerase chain reaction (qRT-PCR) in 0.2% and I+P stimulated HCASMCs. Iman and Conover ranked nonparametric analysis followed by *t* test, n=3 biological replicates. **P<0.05. **E**, Representative FACS histogram plots depicting EdU uptake in siCtrl, siSMILR, and siCENPF treated HCASMCs. Gate represents EdU+ cells. **F**, Bar chart showing mean changes of EdU incorporation in siCtrl, siSMILR, and siCENPF treated cells. Iman and Conover ranked nonparametric analysis followed by *t* test, n=3 biological replicates, *P<0.05.

With arterial and venous SMCs differing significantly, we sought to further investigate the role of *SMILR* in relation to atherosclerosis by validating its mode of action in HCASMCs. First, we confirmed incorporation of EdU in HCASMCs stimulated with IL1-PDGF. This significantly upregulated proliferation, although as previously described (^17^, Figure 6A and 6B), the proliferative capacity of HCASMCs are significantly less than that observed in the HSVSMCs. Nevertheless, with IL1-PDGF induced proliferation in the HCASMCs, we also identified by qRT-PCR significant increases in *SMILR*, *CENPF*, *MKI67*, *AURKB*, and *CDC20* transcripts (Figure 6C and 6D). Importantly and similar to that observed in the HSVSMCs, knockdown of *SMILR* and *CENPF* in HCASMCs (Online Figure XIV) resulted in reduction in proliferation (Figure 6E and 6F). Taken together, this suggests that although arterial and venous SMCs differ significantly, *SMILR*’s mechanism of action remains consistent.

**Figure 7.**
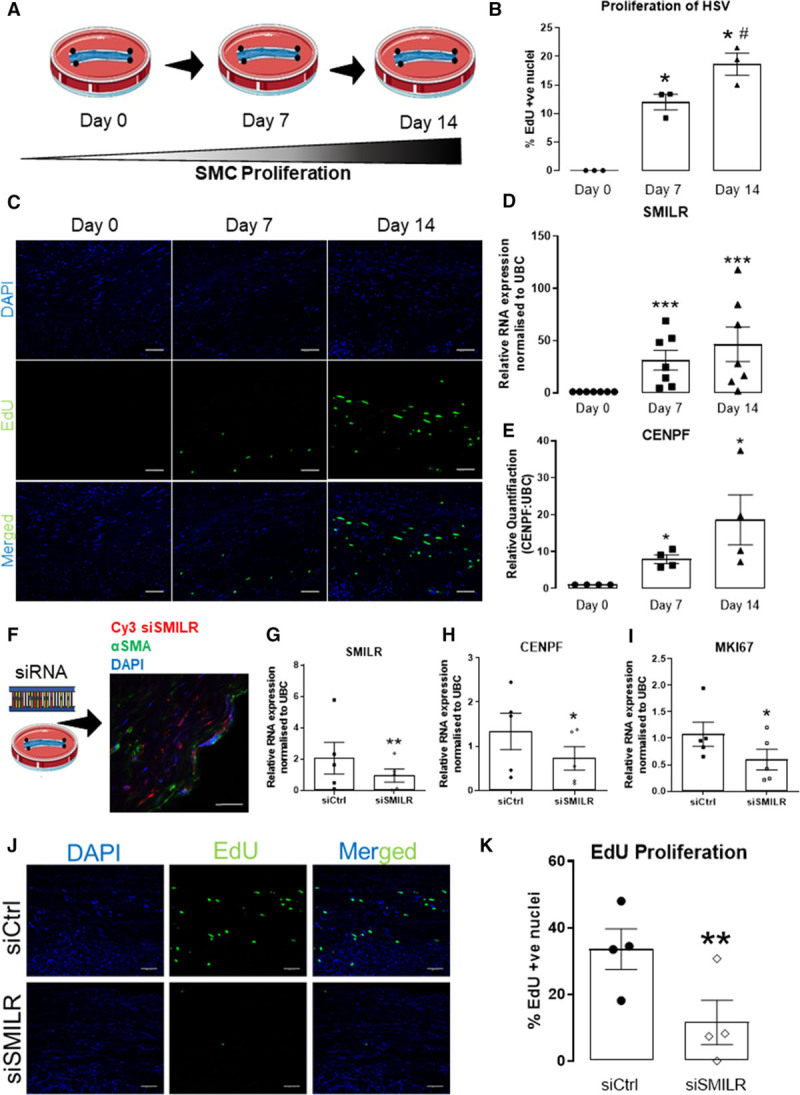
**SMILR modulates the proliferation of the ex vivo human saphenous vein (HSV) organ culture.** **A**, Graphical representation of ex vivo HSV proliferation model. **B**, Quantification of EdU +ve nuclei in the media of HSV in culture expressed as % of EdU +ve/DAPI (4′,6-diamidino-2-phenylindole) +ve nuclei. #*P*<0.05, *P<0.0, * vs day 0, # vs day 7 using Iman and Conover ranked nonparametric analysis followed by 1-way ANOVA (n=3 biological replicates per time point). **C**, Representative images of HSV in culture stained for EdU (green) and with DAPI (blue) at day 0, 7, and 14 (n=3 biological replicates per time point). SMILR (**D**) and CENPF (centromere protein F; **E**) expression determined by quantitative real-time polymerase chain reaction (qRT-PCR) analysis at day 0, 7, and 14. *P<0.05 and ***P<0.001 vs day 0 analyzed by 1-way ANOVA (n=7). **F**, **Left**: Graphical representation of the model of HSV siSMILR intervention; **Right**: Representative image of Cy3-labeled *SMILR* siRNA localization in HSV at 3 d post-siSMILR intervention; (*Continued* )**Figure 7 Continued.** the section is costained with DAPI (blue) and for α-smooth muscle actin (α-SMA, green) at ×60 magnification. Relative quantification of (**G**) *SMILR*, (**H**) CENPF, and (**I**) MKI67 (marker of proliferation Ki-67) expression in HSV after siSMILR intervention at day 7 normalized to UBC (ubiquitin C). *P<0.05, **P<0.01 by paired 2-tailed Student *t* test, n=5 biological replicates. **J**, Representative images of HSV post-siSMILR intervention stained for EdU (green) and with DAPI (blue). **K**, Mean±SEM of EdU +ve nuclei in the media of HSV after siSMILR intervention expressed as % of EdU +ve/DAPI +ve nuclei (n=3 biological replicates). Values are *P<0.05; vs siCtrl using Iman and Conover ranked nonparametric analysis followed by Student *t* test.

We also assessed the *SMILR*: *CENPF* axis in the context of vSMC proliferation associated with vein graft disease. Hereto, we used an ex vivo HSV model,^36,37^ which is associated with time-dependent SMC proliferation, migration, and formation of neointima over 14 days in culture (Figure 7A).^38^ We first validated this approach by monitoring EdU incorporation at 0, 7, and 14 days and found significant increases (Figure 7B and 7C). Thus, we hypothesized that *SMILR* expression may be regulated during the culture period. Accordingly, saphenous veins were cultured for 0, 7, or 14 days and the expression of *SMILR*, *CENPF*, and the downstream cell cycle-associated targets assessed by qRT-PCR. When compared with day 0 control, *SMILR* expression was increased 28±13- (*P*<0.05) and 53±19-fold (*P*<0.01), respectively, at day 7 and 14 (Figure 7D). We also identified a time-dependent increase in *CENPF* expression to 8±1- (*P*<0.05) at day 7 and 19±7-fold (*P*<0.05) at day 14 (Figure 7E). Similar to the qRT-PCR data obtained in Figure 6E, we are able to detect using immunohistochemistry increases in CENPF positive cells in the medial layer from 30% at day 0% to 51% at day 7 (Online Figure XV). Concordantly, expression of other *SMILR* downstream targets within the cell cycle network, namely *AurKB*, *BUB1B*, *MKI67*, and *CDC20*, were upregulated at day 7 (22±8-, 11±3-, 22±8-, and 18±7-fold change, respectively) and day 14 (41±14-, 34±14-, 75±29-, and 50±31-fold change, respectively; Online Figure XVI). Overall, these data suggest that *SMILR* expression and its downstream network has a strong association with pathological remodeling in human ex vivo vein grafts.

We then sought to manipulate *SMILR* expression in the ex vivo saphenous vein graft to assess the clinical relevance and therapeutic potential. We, therefore, used a novel siRNA approach within the clinically relevant time window of an initial 30 minutes (clinical window from harvesting of the saphenous vein to grafting) before culture in which to attempt to knockdown *SMILR*. Cy3-tagged siSMILR was first used to visualize the successful infiltration of the siRNA into the vein (Figure 7F). Because of the limitations of the longevity of siRNA chemistry, by day 14 the siSMILR effects were found to be diminished (Online Figure XVII). In veins with siRNA intervention leading to a significant decrease in *SMILR* levels assessed at day 7 (Figure 7G), we also observed significant decreases in *CENPF* and *MKI67* mRNA expression (Figure 7H and 7I). Finally, quantified proliferation by EdU incorporation in the cultured vein revealed a strong reduction from 28.7±5.3% EdU +ve/DAPI +ve nuclei in control conditions to 5.2±2.6% with *SMILR* knockdown (Figure 7J and 7K; *P*<0.01).

## Discussion

Aberrant growth of vSMCs is a common and functionally important mechanism, which may ultimately contribute to the cause of numerous cardiovascular diseases.^4^ Although the general mechanism of cell cycle regulation is well established,^39^ cell-enriched regulators such as lncRNA are not at all well defined in terms of expression, association, and mechanism, which is crucial for the successful development of targeted therapeutics and improved knowledge of how the human transcriptome can impact physiological and pathological pathways. Here, we identify the mechanism and downstream network of the vSMC-enriched human lncRNA, *SMILR*, and demonstrate its therapeutic potential in the ex vivo HSV model (Figure 7). This has the potential to not only enhance our understanding of atherogenesis, neointimal hyperplasia, and plaque formation but also provides a clear therapeutic target for future investigation in a broad range of cardiovascular diseases.

The human genome contains a wide range of lncRNAs that are dynamically expressed in a temporal and cell-specific manner. These lncRNAs can influence the level and spatial distribution of many proteins and mRNAs to control key aspects of cellular function. LncRNAs have previously been shown to modulate cell cycle, primarily in cancer cell lines.^40,41^ Additionally, lncRNAs, such as smooth muscle and endothelial cell-enriched migration/differentiation-associated lncRNA and myocardin-induced smooth muscle lncRNA, inducer of differentiation, have also been previously shown to be influential in cardiovascular diseases and essential in controlling the phenotypic switching of VSMCs to maintain their contractile phenotype (^42, 43^). More recently, the role of MEG3 (maternally expressed 3) in patients with pulmonary arterial hypertension was examined which revealed significantly reduced MEG3 expression levels in patients compared with healthy controls.^44^ In vitro siRNA silencing of MEG3 resulted in increased SMC proliferation and migration while mechanistic investigation revealed that MEG3 regulates the p53 pathway in PASMCs.^45^ Although several lncRNAs have been identified that control key aspects of SMC and EC function, very little is known about their role in atherosclerosis. A key atherosclerotic lncRNA is ANRIL (antisense noncoding RNA in the INK4 locus), which was identified via genome-wide association studies, in which several SNPs (single nucleotide polymorphisms) located within this lncRNA were associated with atherosclerosis. It was later identified that ANRIL regulates gene expression epigenetically through recruiting repressive components of the polycomb complexes 1 and 2 to ANRIL-target gene promoters via Alu-repeats.^46^

Here, we showed that *SMILR* specifically targets the late mitotic pathway in proliferating HSVSMCs and interacts with *CENPF* mRNA and STAU1. Two recent studies have demonstrated that the lncRNAs, SNHG5 (small nucleolar RNA host gene 5), and TINCR (terminal differentiation-induced ncRNA), counteract STAU1-mediated decay to promote the stabilization of specific mRNAs to control tumor cell survival in colorectal cancer and somatic tissue differentiation, respectively.^29,47^ Similar to *TINCR* and its target mRNA PGLYRP3 (peptidoglycan recognition protein 3), *SMILR’s* interaction with *CENPF* mRNA appears to occur independent of STAU1 protein interaction as revealed by RNA fluorescence in situ hybridization. Although we see upregulation of both *CENPF* mRNA and *SMILR* with STAU1 knockdown, we cannot exclusively conclude whether STAU1’s interaction with *SMILR:CENPF* mRNA is regulating *CENPF* at a post-transcriptional and post-translational stage. Additionally, STAU1 may not only affect the levels of CENPF at an RNA and protein levels but also regulate its subcellular localization since STAU1 has been found to be involved in mRNA transport and localization to mediate further translation.^48^

Whether the *SMILR*/*CENPF* interaction is dependent on base complementarity and/or secondary structure is a key future scientific question as the secondary structure of *SMILR* may be crucial for its localization, downstream interactions, and hence function.^49–51^ Also, other mRNAs might be regulated by *SMILR* and STAU1 and sequencing of associated mRNAs may further provide a comprehensive network of interactions in proliferating vSMCs.

Consistent with our findings, previous studies have indicated that STAU1 primarily binds to protein-coding mRNAs of key mediators of cell cycle and that STAU1 expression and function necessarily fluctuates throughout the cell cycle, being highest during the S-phase and rapidly decreasing during mitotic progression.^34^ Additionally, STAU1 overexpression affects mitotic entry and impairs proliferation of transformed cells, therefore, highlighting STAU1-function must be inhibited in a temporally dependent manner during the cell cycle for proper mitotic progression.^34^ With STAU1 being a ubiquitously expressed and a multifunctional protein, lncRNAs may be crucial for providing its cell-specific function and accordingly mediate cell-specific phenotypes. This may also be the case for *CENPF*, which is also ubiquitously expressed and shown to be multifunctional to control mitotic control, transcriptional regulation, and muscle cell differentiation.^52^ Intriguingly, increased levels of CENPF have also been previously associated with increased proliferation in malignant conditions^31^ and associated with a poor prognosis in human cancers.^53,54^ However, the mechanism by which increased CENPF results in increased proliferation is not entirely understood. One possibility is that the role of CENPF in assembling kinetochore structures required for correct chromosome alignment and separation during mitosis is a rate-limiting step for mitotic progression. Taken together, our study, therefore, suggests that *SMILR* may provide such a critical cell-specific regulation of STAU1 and *CENPF* function in human vSMCS to trigger cell cycle progression and proliferation. Further studies are required to dissect mechanistically the consequence of CENPF mRNA regulation by SMILR. Particularly CENPF’s mRNA stability, transport, and translation as well as the intersection of this with the mitotic phenotype that we observe when SMILR levels are reduced.

*SMILR* was previously suggested to function, at least in part, by regulating its neighboring gene, HAS2 (hyaluronan synthase 2),^17^ although HAS2 is located ≈750 kb from SMILR. However, we showed using RNA-seq that HAS2 is downregulated with *SMILR* knockdown but was not affected by *SMILR* overexpression, confirming previous findings.^17^ We also demonstrated the proliferative effects of *SMILR* occur in the cytoplasmic fraction since the siRNA approach used selectively blocked cytoplasmic *SMILR* expression and would, therefore, unlikely involve a direct targeting of the HAS2 gene in the nucleus. Here, we focused on the direct regulation by *SMILR* in the cytoplasm and find effects mediated by a distinct proliferative network, but we cannot rule out a downstream effect in the nucleus due to *SMILR* manipulation, or indeed a further proliferative effect mediated selectively in the nucleus by *SMILR* by an independent mechanism. In particular, we noticed the presence of histone mRNAs among the dysregulated genes from the RNA-seq data. Although histone mRNAs are not within the list of *SMILR* predicted targets, the observed level change at the RNA level could lead to protein level changes and subsequent transcriptional changes.

The upregulation or downregulation of the *SMILR*-axis and its consequential effects on vSMC proliferation could influence numerous cardiovascular diseases. This was apparent in the ex vivo vein graft model in this study and suggests that this can influence neointimal hyperplasia and hence the long-term success of revascularisation of vein graft after coronary artery bypass surgery. Interestingly, we found a similar role for SMILR in HCASMCs and may, therefore, also be involved in atherosclerosis. However, targeting of SMILR may not be beneficial due to the potential reduction in stability and formation of a fibrous cap. Further studies are, however, required to fully understand the influence of the *SMILR*-axis with respect to SMC proliferation in the atherosclerotic environment and hence the susceptibility to plaque rupture and ultimately myocardial infarction and stroke.

Significantly, within a clinically amenable timeframe, siRNA-based gene therapy targeting *SMILR* is sufficient to markedly reduce proliferation in the ex vivo vein model. This excitingly provides a vSMC-specific target, which reduces the possibility of off-target effects in the remainder of the vessel wall, that is, inhibited re-endothelialization. This strongly suggests that such an intervention may reduce vein graft failure rates. Although our studies only show successful knockdown with siRNA for a limited time frame, whether this is sufficient to maintain a long-term antiproliferative effect is something that requires further studies. Nonetheless, other routes of *SMILR*-targeting gene therapy may be required for maximum longevity such as LNA-GapmeR antisense oligonucleotides.^13^ However, antisense oligonucleotides target both nuclear and cytoplasmic fractions of a cell whereas siSMILR only has a cytoplasmic effect (Online Figure VI). Accordingly, the subsequent effects of antisense oligonucleotides knockdown of *SMILR* in the nuclear fraction must be studied to ensure no detrimental effects.

We demonstrate that *SMILR* is a vSMC-enriched lncRNA, essential in the control of cell cycle through binding of *CENPF* mRNA and STAU1. Our studies provide early but compelling evidence that *SMILR* is an exciting and novel target in the treatment of aberrant growth of vascular SMCs, with the potential to significantly reduce the rate of vein graft failure.

## Acknowledgments

We thank Lynne Maquat for her continued advice and to Gregor Aitchison and Yvonne Harcus for technical assistance. Flow cytometry data was generated with support from the QMRI Flow Cytometry and cell sorting facility, University of Edinburgh. We also thank the University of Edinburgh’s CALM facility used for immunofluroscent microscopy. A.H. Baker, A.D. Mahmoud, M.D. Ballantyne, and J. Rodor designed experiments, interpreted data, and wrote the article. A.D. Mahmoud, M.D. Ballantyne, V. Miscianinov, K. Pinel, J. Hung, and J.P. Scanlon performed experiments. K. Pinel, J. Hung, J. Kaczynski, and A.S. Tavares sampled and conducted imaging on patient atherosclerotic plaques. J. Rodor did the RNA-sequencing (RNA-seq) analysis. I. Ulitsky provided pipeline for RNA-seq analysis. G.W. Gould provided guidance for the cell cycle studies. S.J. George provided guidance on ex vivo studies. N.L. Mills, D.E. Newby, and J.C. Sluimer provided guidance on atherosclerosis and clinical studies. A.C. Bradshaw and J.C. Sluimer provided samples for studies. All the authors discussed the data and edited the article.

## Sources of Funding

This work is supported by the British Heart Foundation (PG/16/51/32180 and RG/14/3/30706) and the University of Edinburgh’s BHF Research Excellence Award (RE/13/3/30183). A.H. Baker is supported by the British Heart Foundation Chair of Translational Cardiovascular Sciences (CH/11/2/28733) and European Research Council (EC 338991 VASCMIR).

## Disclosures

None.

## Supplementary Material

## Novelty and Significance

### What Is Known?

- Like other noncoding RNA, long noncoding RNA (lncRNA) can regulate key aspects of smooth muscle cell function.
- LncRNA *SMILR* regulates smooth muscle cell proliferation via an unknown mechanism.
