# Supplementary figures and images for: The Human-Specific and Smooth Muscle Cell-Enriched LncRNA SMILR Promotes Proliferation by Regulating Mitotic CENPF mRNA and Drives Cell-Cycle Progression Which Can Be Targeted to Limit Vascular Remodeling
Source: Circ Res. 2019 Jul 23;125(5):535–51. doi: 10.1161/CIRCRESAHA.119.314876 (PMC6693924; doi:10.1161/CIRCRESAHA.119.314876)

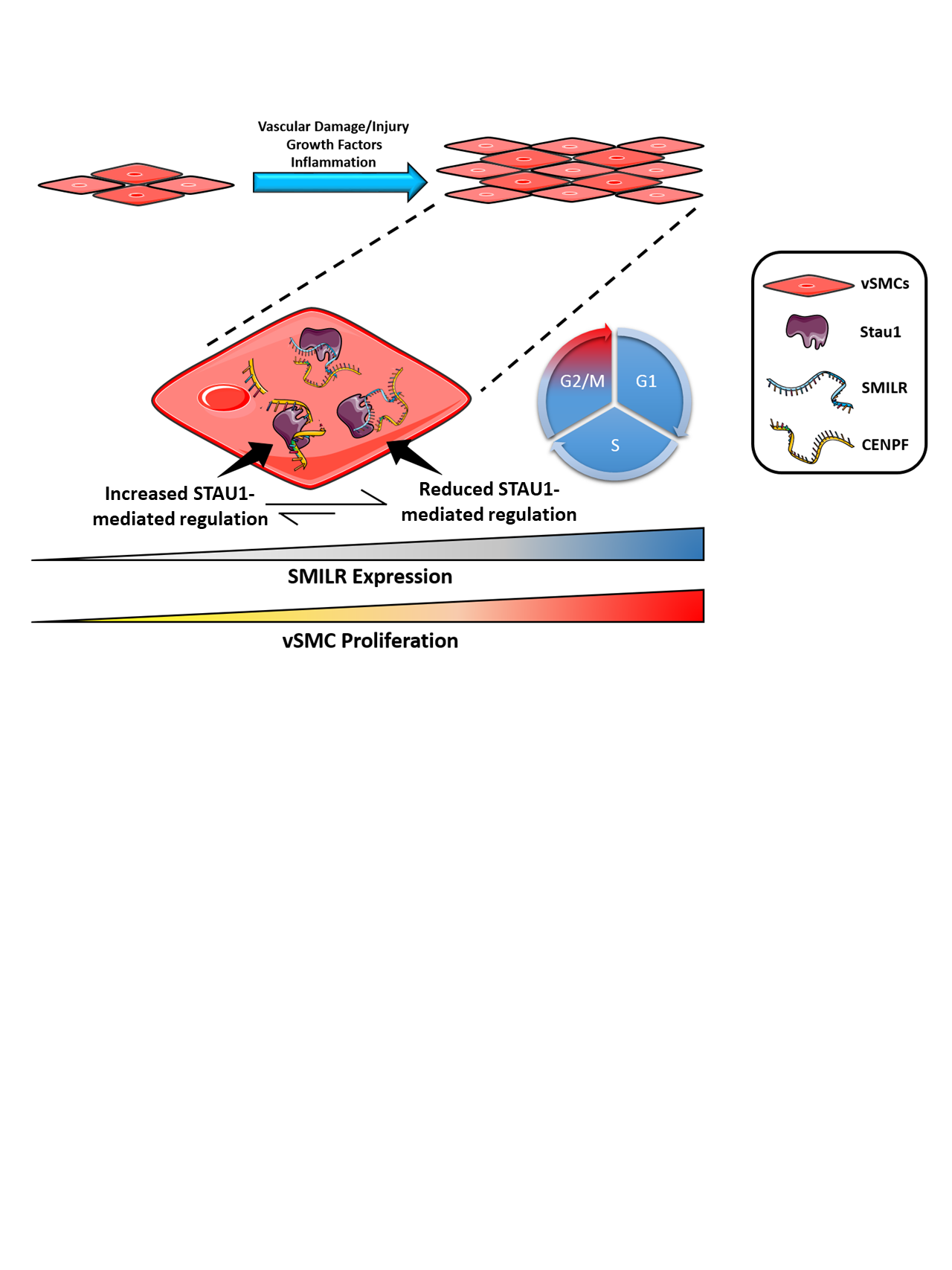

Supplement: Supplementary file 1 [file res-125-535-s001.tif]
