# Supplementary material for: The Human-Specific and Smooth Muscle Cell-Enriched LncRNA SMILR Promotes Proliferation by Regulating Mitotic CENPF mRNA and Drives Cell-Cycle Progression Which Can Be Targeted to Limit Vascular Remodeling
Source: Circ Res. 2019 Jul 23;125(5):535–51. doi: 10.1161/CIRCRESAHA.119.314876 (PMC6693924; doi:10.1161/CIRCRESAHA.119.314876)
Supplement: Supplementary file 2 [file res-125-535-s002.pdf]

Supplementary Material

Title: The Human- and smooth muscle cell-enriched lncRNA SMILR promotes proliferation by regulating mitotic CENPF mRNA and drives cell-cycle progression which can be targeted to limit vascular remodelling.

Authors:
Amira D. Mahmoud[1#], Margaret D. Ballantyne[1#], Vladislav Miscianinov[1], Karine Pinel[1], John Hung[1], Jessica P. Scanlon[1], Jakub Kaczynski[1], Adriana S. Tavares[1], Angela C. Bradshaw[2], Nicholas L. Mills[1], David E. Newby[1], Andrea Caporali[1], Gwyn W. Gould[3], Sarah J. George[4], Igor Ulitsky[5], Judith C. Sluimer[6,1], Julie Rodor[1], Andrew H. Baker[1,6]*.

Affiliations:
1 Queens Medical Research Institute, BHF Centre for Cardiovascular Sciences, University of Edinburgh, UK, EH16 4TJ.
2 Institute of Cardiovascular and Medical Sciences, BHF Cardiovascular Research Centre, University of Glasgow, UK
3 Institute of Molecular Cell and Systems Biology, College of Medicine, Veterinary and Life Sciences, University of Glasgow, UK, G12 8QQ
4 School of Clinical Sciences, University of Bristol, Research Floor Level Seven, Bristol Royal Infirmary, Upper Maudlin St., Bristol BS2 8HW, UK
5 Department of Biological Regulation, Weizmann Institute of Science, Rehovot, Israel
6 Department of Pathology, Maastricht University Medical Center, Maastricht, the Netherlands
# Equal contributions
* Corresponding Author and Lead Contact: Andrew H. Baker, University of Edinburgh, BHF Centre for Cardiovascular Sciences, Queen’s Medical Research Institute, 47 Little France Crescent, Edinburgh UK, EH16 4TJ, Tel: 0131 24 26728, Email: Andy.Baker@ed.ac.uk.

SUPPLEMENTAL METHODS

Human tissues samples

Excess human saphenous vein (HSV) tissue was obtained from patients who have given their written, informed consent prior to undergoing CABG surgery. Carotid plaques were obtained from patients undergoing endarterectomy following an acute and symptomatic neurovascular event. All procedures had local ethical approval (15/ES/0094).

HSV smooth muscle cell culture

Primary HSV derived smooth muscle cells (HSVSMCs) were isolated from medial explants and maintained and treated with IL1-PDGF, dicer substrate siRNA, and lentiviral infections, as previously described by Ballantyne et al. [17].

Dicer substrate siRNA (dsiRNA) mediated transfection

Double stranded dicer substrate siRNA targeting SMILR, STAU1, CENPF, AURKB and siControl were obtained from Integrated DNA Technologies, Leuven, Belgium (See Supplementary Table 3 for sequences). The siControl does not target any sequence in the human, mouse, or rat transcriptomes. Transient transfection was performed with Lipofectamine RNAiMAX (Life Technologies) following the manufacturer's guidelines. Cells were transfected with siRNA and five hours post transfection, cells were quiesced for 48 h in DMEM supplemented with 100 mg/mL of penicillin, 100 IU/mL streptomycin, 2 mM L-glutamine, and 0.2% [v/v] FCS and then stimulated for a further 48 or 96h with fresh 0.2% media containing 10 ng/mL IL1α and 20 ng/mL PDGF-ββ (R&D Systems).

Lentiviral mediated infection

Lentiviral vectors were produced by triple transient transfection of HEK293T cells with a packaging plasmid (pCMVΔ8.74), a plasmid encoding the envelope of vesicular

stomatitis virus (VSVg) (pMDG) (Plasmid Factory, Bielefeld, Germany) and pLNT/SFFV-MCS plasmid employing polyethylenimine (PEI; Sigma-Aldrich, St Louis, USA) as previously described [1]. Lentiviral titres were ascertained by TaqMan quantitative real-time PCR (qRT-PCR) using the following primer/probe sequences: forward, 5’-TGTGTGCCCGTCTGTTGTGT-3'; reverse, 5'- GAGTCCTGCGTCGAGAGAGC-3'; probe, 5'-(FAM)-CAGTGGCGCCCGAACAGGGA- (TAMRA)-3. A confluent monolayer of smooth muscle cells were plated and quiesced for 48 hours prior to being infected with either SMILR_LNT or the FUCCI virus (kind gift from Dr. Alan Serrels, Edinburgh, UK). Following 24 h infection, media was changed to 0.2% media for a further 24h before being stimulated with 10 ng/mL IL1α and 20 ng/mL PDGF-ββ for further 96h.

Inhibition of transcription factors

Growth-arrested HSVSMCs were treated for 1 hour with JSH-23 (NFKB inhibitor 40 µM), 3930 (NFAT inhibitor 50 µM) or SR 11302 (AP1 inhibitor 1 µM) prior to stimulation with IL1 and PDGF for 72 h. Total cellular RNA was isolated and levels of SMILR and positive control MKI67 examined via qRT-PCR.

RNAseq analysis

RNAseq was performed on RNA extracted and DNAse treated samples using the miRNeasy mini kit (Qiagen) obtained from three replicates of SMILR depletion and overexpression samples as well as from “stable” and “unstable” plaque section obtained from 4 independent patients.

Ribosomal-depleted unstranded libraries were prepared by GENEWIZ, Inc. (South Plainfield, New Jersey, USA) and were sequenced with Illumina HiSeq (paired end 2x100bp). Between 34 and 40 million paired reads were obtained for “SMILR” RNAseq to

analyse protein-coding gene differential expression. For the atherosclerosis RNAseq, we obtained between 57 and 72M paired end read, allowing a deeper coverage. Gene quantification (read count and FPKM) was obtained using RSEM (options: -bowtie2 -paired-end) [2], based on GENCODE annotation (Release 25). For both RNA-sequencing experiments the differential expression was performed utilizing edgeR [4]. We considered a threshold of absolute Fold Change >=1.5 and adjusted pvalue<0.05 to identify significant changes between two conditions.

Sample clustering was evaluated using the Principal component analysis (PCA) tool available in DESeq2 on the regularized log transformed data. The 3D plot was obtained using the rgl R package.

The gene ontology analysis was done using topGO [6] on enriched genes over a background of expressed genes (FPKM>2 in at least one condition). Fisher's exact test was used to calculate the p-values.

Both data set are deposited in the Gene Expression Omnibus (GEO) repository, study number GSE120521 for the atherosclerosis RNAseq and GSE117608 for SMILR RNAseq.

Fluorescent Imaging

HSVSMC were transfected with siControl, siSMILR, or siAURKB, and plated at 1 x 104 cells per well in a 6-well plate containing coverslips, before being quiesced and then stimulated with IL-PDGF for 96h. They were then fixed in 4% paraformaldehyde (PFA) for 10min and then permeabilised with 0.1% Triton X for 2-3min before adding Phalloidin-iFluor 488 reagent CytoPainter (Abcam) for 20min and imaged using Andor Revolution XDi spinning disk confocal microscope. HSVSMCs infected with FUCCI virus as described above were also imaged using the Andor Revolution XDi spinning disk confocal microscope.

Flow cytometry

Single-cell suspensions of FUCCI-infected HSVSMCs were fixed for 5 min in 70% Ethanol, followed by an incubation in PBS/1% BSA containing for 1h at 4˚C and then resuspended in PBS for FACs analysis on the BD LSR5 Fortessa Analytic Flow Cytometer. Dot plots were generated using FlowJo software.

Gene expression quantitative RealTime-PCR (qRT-PCR)

For gene expression analysis, cDNA for mRNA analysis was obtained from total RNA using the Multiscribe Reverse Transcriptase (Life technologies, Paisley, UK). qRT-PCR was performed using Power SYBR green (Life Technologies) with custom PCR primers (Eurofins MWG, Ebersberg, Germany, Supplementary Table 2 – primer sequences). Alternatively, qRT-PCR was performed using Universal Taqman Master Mix™ (Applied Biosystems) with Taqman probes (Supplementary Table 4). Ubiquitin C (UBC) were selected as housekeeping genes due to their stability across all groups and conditions studied. Fold-changes were calculated using the 2-ΔΔCt method [7]

vSMCs EdU Incorporation assay

Similar to [1], HSVSMCs proliferation was quantified using EdU according to the manufacturer’s instructions. Briefly, cells were plated and quiesced in 0.2% FCS media for 48 h prior to stimulation. Fresh media was then added with EdU for the times stated. Cells were then fixed in 70% ethanol for EdU FACs analysis. EdU incorporation was quantified using Click-it EdU Proliferation assay with an Alexa Fluor 594 antibody according to the manufacturer's protocol (Life Technologies).

T7-SMILR production and biotinylation

To generate 50pmol of biotinylated lncRNA required for each RNA-Protein Pull-down, 1µg of cDNA is used as a template for T7 RNA polymerase in vitro transcription using T7 RiboMAX Express Large Scale RNA Production System Protocol (Promega) and the T7 primer sequences listed in Supplementary Table 1. The resulting RNA is extracted using the miRNeasy mini kit (Qiagen) following manufacturer's instructions. Commercially available pCp – Desthiobiotin (Jena Biosciences) are then attached to the 3'end of the RNA strand using T4 RNA ligase and Pierce RNA 3' End Desthiobiotinylation kit (Thermo Scientific).

RNA-antisense Probe Pulldown

Antisense DNA probes were designed targeting the full length sequence of SMILR [1] or GFP (used as negative control) using the online designer at https://www.biosearchtech.com/stellaris-designer. Probes mapping to tandem repeat sequences or to homologous genes were excluded. 10 probes of 40bp were generated for SMILR and 5 for GFP (Sequences in Supplementary Table 5). The SMILR probes were split into two sets based on their relative positions along the gene sequence and were accordingly referred to as even or odd capture oligo sets. All probes were biotinylated at the 3'end with an extended 15 atom triethylene glycol spacer arm (IDT).

SVSMCs stimulated with IL-PDGF for 48h were rinsed once with PBS before crosslinking with 2% formaldehyde at 37C for 10min. Crosslinking was quenched with 500mM glycine at 37˚C for 5min and then the cells were washed with ice cold PBS. Subsequently, cells were scraped in ice cold PBS + 0.5% BSA Fraction V and moved into Falcon tubes and centrifuged and pelleted at 1,000g. Thereafter, the supernatant was removed and the pellet was resuspended in scraping buffer. At this point, cells were counted and the resuspended pellet was divided into falcon tubes of 20 million cells each. These were then pelleted again at 2,000g and snap frozen and stored in -80C for subsequent steps.

To prepare lysates for pulldown, cell pellets were quickly thawed in a 37 °C water bath and resuspended in cell lysis buffer (50 mM Tris, pH 7.0, 10 mM EDTA, 1% SDS, and added just before use: dithithreitol (DTT), phenyl-methylsulphonyl fluoride (PMSF), and RNAse inhibitor) on ice for 10 min. Cell lysates were then diluted in double the volume of hybridization buffer (500 mM NaCl, 1% SDS, 100 mM Tris, pH 7.0, 10 mM EDTA, 15% formamide, and added just before use: DTT, PMSF, protease inhibitor, and RNAse inhibitor. Probes (50 pmol each) were added to lysate, which was mixed by end-to-end rotation at 4°C overnight. Streptavidin-magnetic C1 beads were washed three times in cell lysis buffer, 500µl of beads was added to hybridization reaction and the whole reaction was mixed for 30 min at 37°C. Beads–biotin-probes–RNA adducts were captured by magnets (Invitrogen) and washed five times with a wash buffer (2×SSC, 0.5% SDS, fresh PMSF added). After the last wash, buffer was removed and beads were resuspended in 200µl RNA proteinase K buffer (100 mM NaCl, 10 mM Tris, pH 7.0, 1 mM EDTA, 0.5% SDS) with fresh 1 mg ml−1 proteinase K added (Ambion). After incubation at 50°C for 45 min, followed by boiling for 10 min, RNA was spiked with 75ng of total C. elegans RNA to use AMA1 as a reference gene as previously described [1] was isolated using the chloroform isoamyl approach. Eluted RNA was subject to DNase treatment (TURBO DNase-free kit, Ambion) followed by qRT–PCR for the detection of enriched transcripts.

Predicting SMILR-RNA interactions

Sum energy based ranking method was used to predicate SMILR and RNA interactions [8]. We first filtered the top100 list based on their expression levels in IL1-PDGF stimulated vSMCs in our control RNAseq samples (i.e. siControl and Null LNT). Genes falling below the expression threshold of an FPKM < 1 were discarded. We then considered all the remaining expressed genes and filtered through those that were found to be modulated following

SMILR knockdown or overexpression. However, as we know that SMILR's upregulation and function is dependent on IL1-PDGF stimulation, we further filtered the genes that were differentially regulated with IL1-PDGF from 0.2% based on RNAseq data from Ballantyne el al. 2016, [1].

SMILR-Protein pulldown

Streptavidin magnetic beads are used to capture the biotinylated RNA target, after which, 20µg of smooth muscle cell lysate are incubated with the magnetic bead/RNA complex. This was performed using the Pierce Mag RNAProtein Pulldown kit (Thermo Scientific). Stringent washes then removed any non-specific RNA-protein interactions and the remaining lncRNA-binding proteins are eluted using a non-denaturing biotin elution buffer for downstream analysis, i.e. liquid chromatography mass spectrometry (LC-MS).

Mass spectrometry

In excess of 2µg of eluted proteins were separated by polyacrylamide gel electrophoresis (PAGE) and visualised using a Coomassie stain. The protein bands are excised before undergoing an in-gel trypsin digestion for LC-MS. LC-MS is performed on a Q exactive mass spectrometer coupled to an Ultimate 3000 RSLCNano System (Dionex; Thermo Fisher Scientific). Data analysis was then performed using the MaxQuant software platform (ver. 1.5.2.8) and searches were conducted against a Human Uniprot database. Subtractive proteomics was used to identify proteins only detected in SMILR pulldowns and only proteins with at least 2-fold enrichment (LFQ) in SMILR pulldowns versus GFP and/or background (beads only pulldown), with at least 2 unique peptides, were considered for further downstream analysis and validation.

STAU1 Pulldowns

SVSMCs stimulated with IL-PDGF for 48h were rinsed once with PBS before crosslinking with 2% formaldehyde at 37˚C for 10min. Crosslinking was quenched with 500mM glycine at 37˚C for 5min and then the cells were washed with ice cold PBS. Subsequently, cells were scraped in ice cold PBS + 0.5% BSA Fraction V and moved into Falcon tubes and centrifuged and pelleted at 1,000g. Thereafter, the supernatant was removed and the pellet was resuspended in scraping buffer. At this point, cells were counted and the resuspended pellet was divided into falcon tubes of 20 million cells each. These were then pelleted again at 2,000g and snap frozen and stored in -80C for subsequent steps.

Cell pellets were thawed at 37˚C and resuspended in cell lysis buffer (50 mM Tris, pH 7.0, 10 mM EDTA, 1% SDS, and added just before use: dithithreitol (DTT), phenyl-methylsulphonyl fluoride (PMSF), and RNAse inhibitor) on ice for 10 min. 13. Samples were then precleared with Dynabeads G (ThermoFisher) at 4˚C for 1 h using an inversion shaker that rotates the tubes end-to-end.

Then using a DynaMag-2 (ThermoFisher), beads were collected against the magnet and the supernatant was split evenly into two fresh Eppendorf. In each of the two tubes, fresh Dynabeads G, cOmplete Mini, EDTA-free Protease Inhibitor Cocktail tablets (Roche) and RNAse inhibitors were added, and then either 4µg of mouse IgG (Abcam) or Stau1 antibodies (Santa Cruz Biotechnology) were added. The samples were then rotating at 4˚C for 2-3 h before being washed using the DynaMag-2 5 times using NET-2 buffer (50 mM Trsi-HCl pH 7.4, 200 mM NaCl, 0.05% NP40, and cOmplete Mini, EDTA-free Protease Inhibitor Cocktail tablets (Roche), and RNAse inhibitors). After the last wash, buffer was removed and beads were resuspended in 200µl RNA proteinase K as described previously. After incubation at 50°C for 45 min, followed by boiling for 10 min, RNA was isolated using the

chloroform isoamyl approach. Eluted RNA was subject to DNase treatment (TURBO DNase-free kit, Ambion) followed by qRT–PCR for the detection of enriched transcripts.

Western blotting

Protein lysates were obtained by lysis of stimulated HSVSMC with RIPA buffer. For STAU1 western blots, membranes were incubated with primary mouse monoclonal antibody for STAU1 (Santacruz, Stau1 D-5, 1:500) at 4°C overnight. Alpha tubulin (Abcam, ab18251, 1:2000) was utilised as a loading control. After washing, membranes were incubated with the appropriate Licor IRDye® 800 secondary antibody (1:20,000) at room temperature for 1 h. Following additional washing protein levels were visualised via Licor.

In Vivo Studies Atherosclerosis Studies: Patients, Imaging and Sampling

Similar to Ballantyne et. al. [1] patients with symptomatic carotid artery stenosis scheduled to undergo carotid endarterectomy were recruited from neurovascular clinics at the Royal Infirmary of Edinburgh to undergo separate [18F]-fluoride and [18F]-fluorodeoxyglucose ([18F]-FDG) positron emission tomography [9] combined with computed tomography (CT) scans with the use of a hybrid scanner (Biograph mCT, Siemens Medical Systems, Erlangen, Germany). Both of these tracers have been used by our group and others for plaque imaging and highlight high-risk actively calcifying [9] and inflamed or hypoxic atherosclerotic plaques and were administered as previously described in [1, 9]. PET tracer uptake was quantified using an OsiriX workstation (OsiriX version 3.5.1 64-bit; OsiriX Imaging Software, Geneva, Switzerland). PET/CT image data were reviewed for evidence of tracer uptake, image quality and registration. The CT angiogram was examined to establish plaque presence, location and characteristics. Regions of interest were then drawn on three adjacent 3-mm PET slices to incorporate the internal carotid artery plaque. Three ROI were

then drawn around adjacent healthy portions of carotid artery and the lumen of the SVC to derive control values for "normal" arterial uptake and the blood pool respectively. Arterial standardized uptake values (SUV) were recorded and also indexed to blood pool activity thus giving a target-to-background-ratio (TBR).

At the time of surgery, plaques were collected immediately following excision and photographed. Two-millimeter diameter core biopsy specimens for RNA analysis were taken from regions of focally high uptake on PET and from normal tissue at the periphery of the endarterectomy specimen. These, along with the main specimen, were immediately frozen and placed in an -80°C fridge for subsequent batch analysis.

RNA in situ hybridization of human carotid atherosclerotic plaques

Formalin-fixed paraffin embedded (FFPE) tissue slides were deparaffinised in xylene and ethanol (100%, 96%, 70%) solutions at room temperature ending up in RNase-Free water followed by RNase-Free PBS wash. The tissue was then treated with 1:1000 Proteinase K (miRCURY LNA miRNA ISH Buffer Set (FFPE), Qiagen) diluted in RNase-Free PBS for 5 minutes at 37C. After serial RNase-Free PBS washes the LNA SMILR and scrambled RNA probes (IDT) were applied onto the tissue, diluted in 1x Formamide-free miRNA ISH buffer (miRCURY LNA miRNA ISH Buffer Set (FFPE); Qiagen) at 60 nM final concentrations. The tissue slides were hybridized in 55C for 1 hour. The tissue slides were then washed in 5x SSC buffer (made up with RNase-Free water from 20x SSC, ThermoFisher Scientific) at 55C followed by one room temperature wash. The tissue was then blocked with 1x blocking buffer (made up with maleic acid from 10x blocking reagent, Roche) for 1 hour followed by the incubation with Anti-Digoxigenin-AP diluted 1:500 in 1x blocking buffer (Fab fragments, Roche) over night. The tissue slides were then washed three times with RNase-Free PBST and incubated with the detection solution prepared by diluting NBT/BCIP tablet (Roche) in

10 mL of RNase-Free water with 40 µL of Levamisole solution (SP-5000-18). The reaction was stopped after 30-40 minutes, the tissue was washed with RNase-Free PBS and counterstained with Nuclear Fast Red solution (Sigma) for 5 minutes. The tissue was then dehydrated (ethanol 70%, 80%, 95%, 100% 20 seconds each followed by 2 minutes in xylene) and mounted with xylene-based mounting medium. Zeiss Axio Scan.Z1 Slide Scanner was used to take the images, which were analyzed with Zen software.

RNA-Fluroescent in-situ hybridization

Custom RNA-FISH tiled probe sets were generated to all exons of SMILR [1] and CENPF (Thermo Fisher Scientific). RNA-FISH was performed according to manufacturer's instructions (ViewRNA™ cell FISH) with minor changes. HSVSMCs were grown on 16-mm coverslips to 80% confluency, washed in PBS and fixed in 4% paraformaldehyde with 1% glacial acetic acid. Following detergent QS permeabilisation and 1:6000 protease digest, coverslips were incubated with a combination of SMILR and CENPF probe sets. Probe set buffer was used as a negative control and specificity of probes were tested under siSMILR and siCENPF conditions. Following probe hybridisation, cover slips were incubated with branched tree technology pre amplifier for 1h and then with the amplifier for 30 min. Coverslips were then mounted onto glass slides using VECTASHIELD Antifade Mounting Medium with DAPI (Vector Laboratories) and imaged using Andor Revolution XDi spinning disk confocal microscope.

Cellular Fractionation

RNA fractionation was performed according to the manufacturer's instructions provided in the PARIS™ Kit (Thermo Fisher) on HSVSMCs that were plated at 1 x 10^5 cells per well in a 6 well culture dish and transfected with siRNA and stimulated as described above.

Human Saphenous Vein (HSV) Organ Culture

HSV surplus segments were collected as detailed previously [18]. The vein was placed in PBS. The adventitial layer was removed, and the vein was opened longitudinally and cut transversely into three 5-10 mm segments. Vein segments were pinned down with minutien pins on a Sylgard coated dissection dish with the luminal surface facing upward for up to 14 days in DMEM supplemented with 100 mg/mL of penicillin, 100 IU/mL streptomycin, 2 mM L-glutamine, and 10% [v/v] fetal calf serum [FCS] (and with 10 µM EdU for the medial proliferation assay) and cultured separately. The organ culture growth media was changed every 2 days. At day 0 and after 7 and 14 days of culture, the vein segments were washed in PBS and snap frozen for subsequent RNA extraction or fixed in 4% PFA for histology.

Proliferation in HSV Organ Cultures

Paraffin wax-embedded sections were assessed by Click-iT® EdU Alexa Fluor® 488 In Vivo Imaging Kit. Formalin-fixed paraffin embedded (FFPE) sections (3µm) were deparaffinised in 3x clearene (Leica Biosystems) and 3x 100% ethanol washes, 5 minutes each at room temperature ending up in ultrapure water followed by PBS wash. Tissue was then washed in 3% BSA/PBS and permeabilized with 0.5% Triton/PBS. After serial 3% BSA/PBS washes, tissue was incubated with a Click-iT® EdU reaction cocktail for 30 minutes in the dark at room temperature. Slides were then washed in 3% BSA/PBS, followed by PBS and incubated with 1:700 DAPI for 15 minutes. Slides were then washed in PBS and mounted with PermaFluor™ Aqueous Mounting Medium (Thermo Scientific™). Quantification of the EdU incorporation was carried out by calculating the percentage of EdU-positive cells in the media.

siRNA Intervention in HSV Organ Culture

HSV segments cut in equal pieces of approximately 1 cm^2 were bathed in PBS containing 25µM siSMILR, cy3-siSMILR and scrambled siRNA control for 30 minutes in 24-well plate. After 30 minutes of incubation, the vein segments were washed with PBS and pinned down with minutien pins on a Sylgard coated dissection dish with the luminal surface facing upward for up to 14 days in DMEM supplemented with 100 mg/mL of penicillin, 100 IU/mL streptomycin, 2 mM L-glutamine, and 10% [v/v] fetal calf serum [FCS] (and with 10 µM EdU for the medial proliferation assay) and cultured separately. The organ culture growth media was changed every 2 days. At day 3, day 7 and day 14 of culture, the vein segments were washed in PBS and snap frozen for subsequent RNA extraction or fixed in 4% PFA for histology.

Cy3-siSMILR and αSMA co-staining

At day 3 post-Cy3-siSMILR treatment, HSV segment was cryopreserved in OCT. 5 µm tissue sections were cryosectioned and fixed for 15 minutes in 4% PFA made up in PBS. Sections were washed 3 times in PBS, blocked and permeabilized in PBS containing 5% goat serum and 0.1% Triton X-100 for 1 h, and incubated with primary antibody, anti-αSMA (DAKO, M0851, 1:100), diluted in blocking solution overnight at 4°C. Sections were subsequently washed in PBS containing 0.1% Triton X-100, incubated in the secondary antibody, goat anti-Mouse IgG (H+L), Alexa Fluor® 488 (Invitrogen, A28175, 1:500) for 2 h, and washed with PBS three times. Sections were mounted in ProLong™ Gold Antifade Mountant with DAPI (Invitrogen, P36935). Images were acquired using Zeiss Cell Observer spinning disc confocal microscope. Cy3-siSMILR signal was visualized at 555 wavelength.

Quantification of CENPF protein level in HSV media

Human saphenous veins were harvested and fixed in 4% buffered formalin for paraffin embedding, before being sectioned (5 μm) and mounted onto poly-L-lysine coated slides. The tissue sections were deparaffinised and rehydrated by washing in Xylene for 5mins, followed by sequential washes in 100%, 96% and 70% ethanol respectively prior to antigen retrieval in sodium citrate buffer (10mM, pH6.0). Sections were washed in TBS-T and blocked in 3% goat serum in TBS for 1hr at RT, and then stained with rabbit polyclonal anti-CENPF antibody (Abcam, ab5:1:50), or a rabbit isotype control IgG at equivalent concentrations to primary antibody (Abcam, ab172730: 1:33). Following incubation, the section were washed in TBS-T and incubated with HRP anti-rabbit secondary antibody (Sigma, A0545: 1:500). Detection was carried out using 3,3'Diaminobenzidine (DAB) (Vector Laboratories, SK-4105) and a haematoxylin counter-stain applied, before being rehydrated and mounted in Pertex mounting media. An Axioscan slidescanner was used to image the sections, using Zen software. All settings for the Axioscan and software were optimised and then maintained for each set of experiments, so that sections can be compared accurately.

Analysis of staining was performed on QuPath 0.1.2 software. Positive cell detection was utilised, which automatically detects cell nuclei, and whole cell positive CENPF staining was determined by setting a threshold for CENPF detection using positive and IgG controls to confirm threshold limits. The region of interest (medial layer) was selected along the whole vein section and the number of CENPF-positive cells expressed as a percentage of total cells in that region. For each vein, at least 500 cells per section were counted, to give an accurate percentage reading.

Statistical Analysis

Statistical analysis was performed according to figure legends. Data are expressed as mean ± SEM. For samples sizes ≥ 5 normal distribution was determined by Shapiro-Wilk test followed by student t test or ANOVA. For each biological replicate, control and test conditions were set up and accordingly analysed as pairs. Normal distribution cannot be determined on small samples sizes and samples with $n<5$ were assumed to be not normally distributed and subjected to Iman and Conover non parametric ranking followed by students t test or ANOVA. Statistical significance is denoted by a P value of less than 0.05 (Graph Pad Prism version 5.0).

Graphical Image Construction

Graphical images were generated using basic components from ElSevier Medical Art.

Supplemental Methods References

1. Ballantyne MD, Pinel K, Dakin R, Vesey AT, Diver L, Mackenzie R, Garcia R, Welsh P, Sattar N, Hamilton G, Joshi N, Dweck MR, Miano JM, McBride MW, Newby DE, McDonald RA and Baker AH. Smooth Muscle Enriched Long Noncoding RNA (SMILR) Regulates Cell Proliferation. *Circulation*. 2016;133:2050-65.
2. Li H. A statistical framework for SNP calling, mutation discovery, association mapping and population genetical parameter estimation from sequencing data. *Bioinformatics*. 2011;27:2987-2993.
3. Love MI, Huber W and Anders S. Moderated estimation of fold change and dispersion for RNA-seq data with DESeq2. *Genome Biology*. 2014;15:550.
4. Robinson MD, McCarthy DJ and Smyth GK. edgeR: a Bioconductor package for differential expression analysis of digital gene expression data. *Bioinformatics*. 2010;26:139-140.
5. Rajkumar AP, Qvist P, Lazarus R, Lescai F, Ju J, Nyegaard M, Mors O, Børglum AD, Li Q and Christensen JH. Experimental validation of methods for differential gene expression analysis and sample pooling in RNA-seq. *BMC Genomics*. 2015;16:548.
6. Rahnenfuhrer AAaJ. topGO: Enrichment Analysis for Gene Ontology. *R package version 22702016*. 2016.
7. Livak KJ and Schmittgen TD. Analysis of Relative Gene Expression Data Using Real-Time Quantitative PCR and the $2^{-\Delta\Delta CT}$ Method. *Methods*. 2001;25:402-408.
8. Terai G, Iwakiri J, Kameda T, Hamada M and Asai K. Comprehensive prediction of lncRNA–RNA interactions in human transcriptome. *BMC Genomics*. 2016;17:12.
9. Joshi NV, Vesey AT, Williams MC, Shah ASV, Calvert PA, Craighead FHM, Yeoh SE, Wallace W, Salter D, Fletcher AM, van Beek EJR, Flapan AD, Uren NG, Behan MWH, Cruden NLM, Mills NL, Fox KAA, Rudd JHF, Dweck MR and Newby DE. 18F-fluoride positron emission tomography for identification of ruptured and high-risk coronary atherosclerotic plaques: a prospective clinical trial. *The Lancet*. 2014;383:705-713.

A

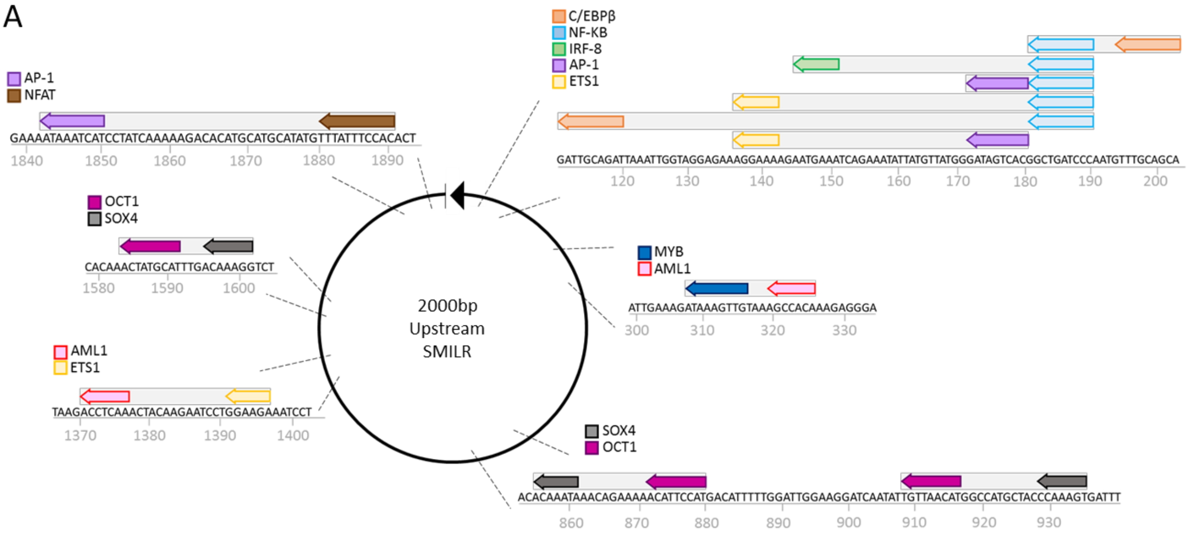

B

Transcription factor name	Number of binding sites	Activated by PDGF-BB signalling	Activated by IL1α signalling
NF-KB*	1	Yes	Yes
CEBPβ	2	Yes	Yes
ETS1	3	Yes	Yes
AP1 *	3	Yes	Yes
NFAT *	1	Yes	Yes
IRF8	1	Yes	No
MYB	1	Yes	No
OCT1	3	No	No
SOX4	3	No	No
AML	2	Yes	No

* Inhibitors commercially available

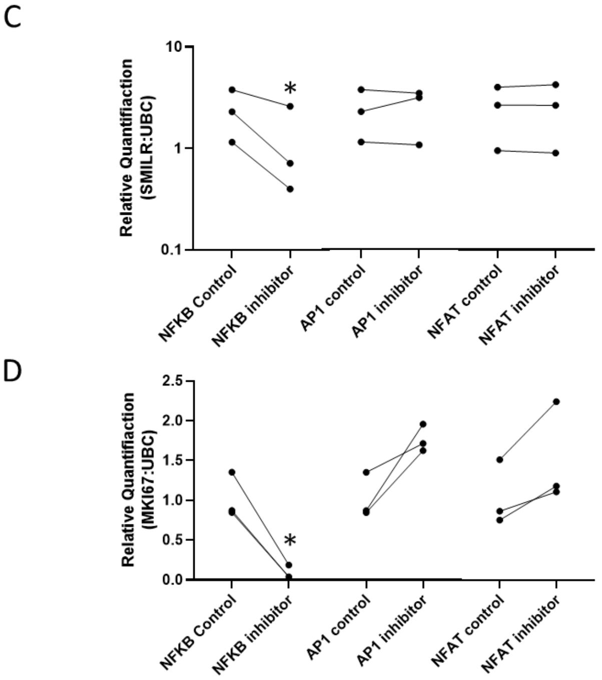

Supplemental Figure I: Characterisation of SMILR promoter region. (A) Predicted transcription factor binding sites within the 2000bp sequence upstream of SMILR's transcription start site. (B) Transcription factors previously shown to be activated by IL1a and/or PDGF-BB. * indicates commercially available inhibitors. (C) Effects of NF-KB, AP1, and NFAT inhibition on SMILR expression. * = $p < 0.05$, nonparametric Iman and Conover ranked analysis and students t-test, each line represents a biological replicate pair (n=3). (D) Effects of NF-KB, AP1, and NFAT inhibition on MKI67 expression. * = $p < 0.05$ nonparametric Iman and Conover ranked analysis and students t-test, each line represents a biological replicate pair (n=3).

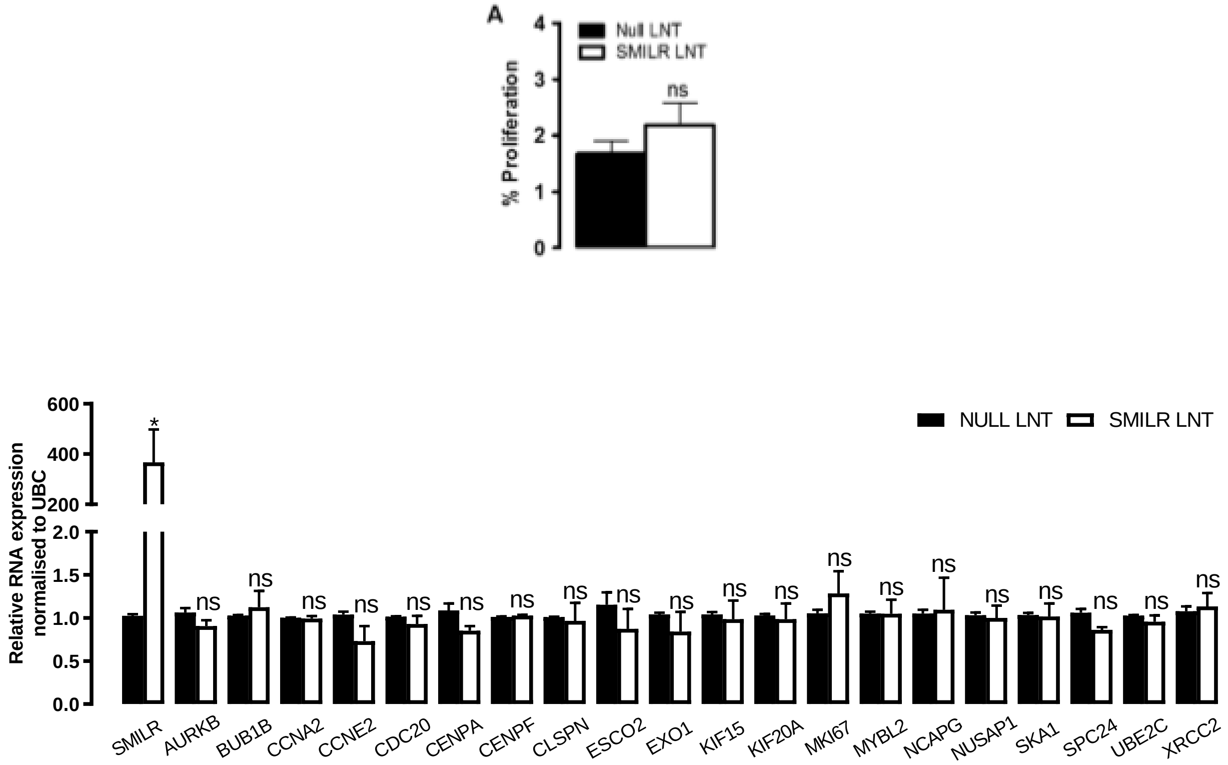

Supplemental Figure II. Effects of SMILR overexpression on quiesced vSMCs. (A) Percentage proliferation of quiesced vSMCs of Null LNT versus SMILR LNT by EdU incorporation and FACs. ns = not significant by paired t-test of n = 3 biological replicates. (B) Bar charts of the relative quantification of 20 cell cycle associated genes between Null LNT versus under quiesced conditions. ns = not significant, * = $p < 0.05$ by nonparametric Iman and Conover ranked analysis and students t-test, n = 3 biological replicates.

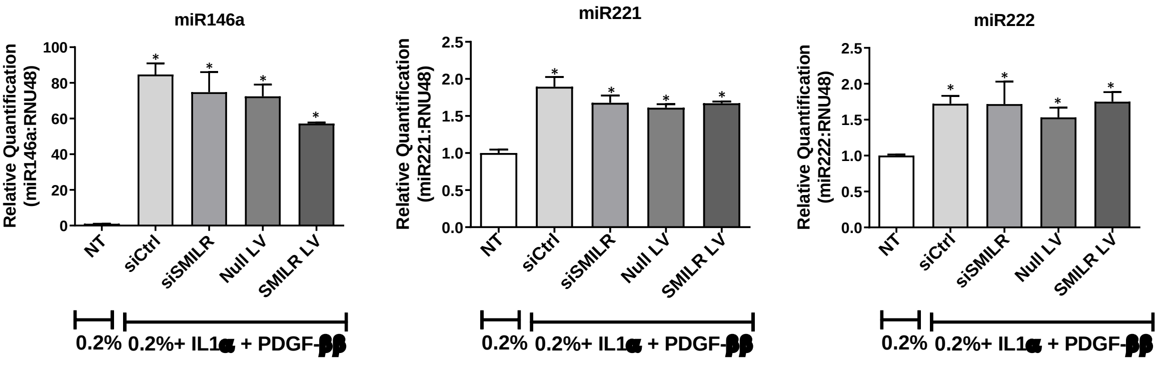

Supplemental Figure III. IL1-PDGF induced expression of mir146a, mir221, and mir222.

Analysis of miR-146a, miR-221 and miR-222 expression normalised to RNU48 via qRT-PCR of samples used for RNAseq analysis from one biological replicate in a technical triplicate.

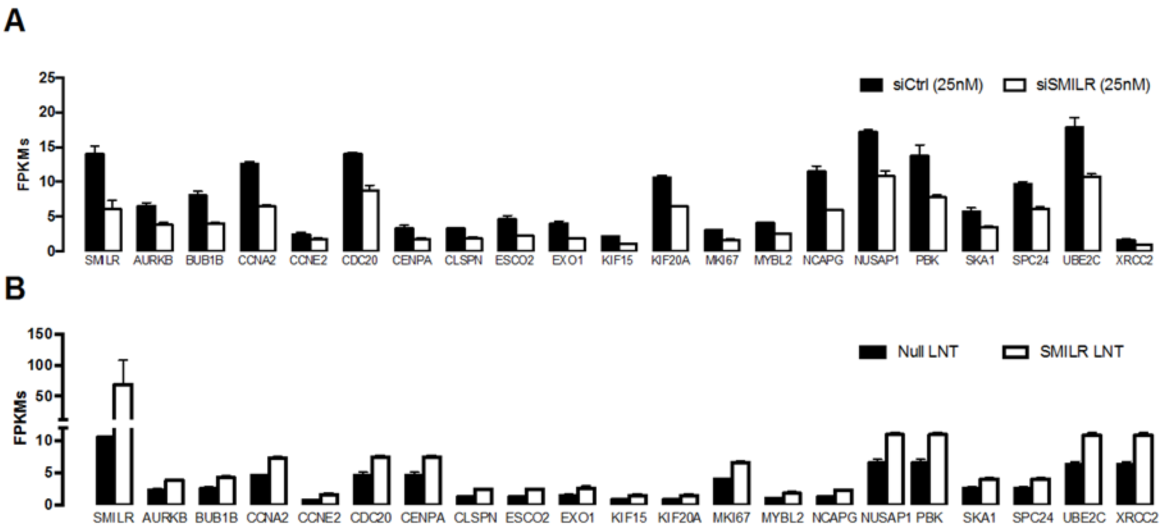

Supplemental Figure IV. FPKM values for the top 20 dysregulated cell division associated genes with (A) siControl (siCtrl) and siSMILR and (B) Control lentivirus (Null LNT) and SMILR lentivirus (SMILR LNT) across 3 technical replicates of one biological replicate.

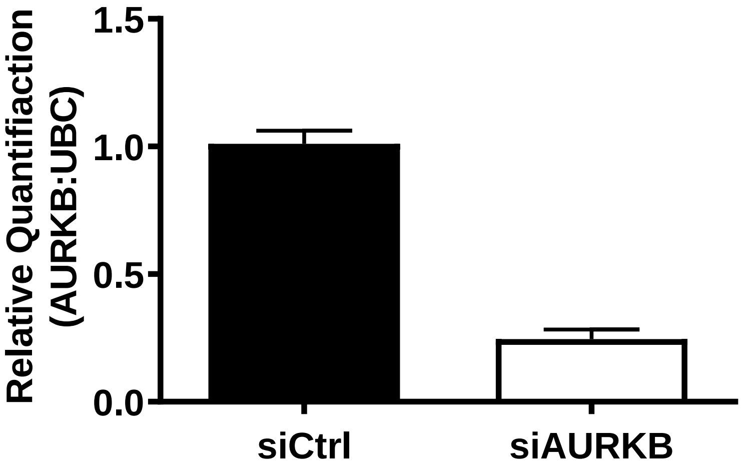

Supplemental Figure V. Relative quantification of AURKB knockdown by qRT-PCR. n = 3 biological replicates.

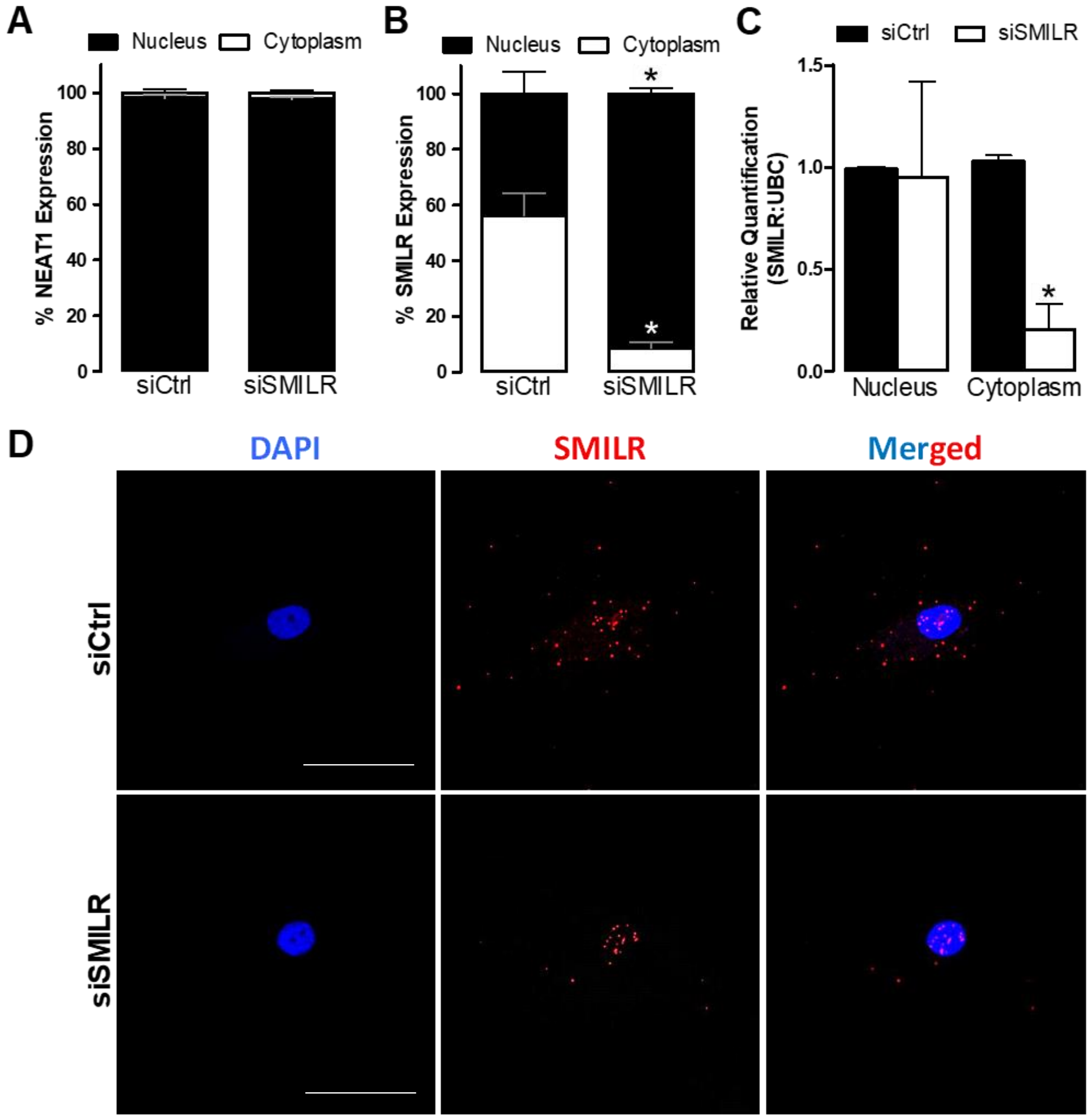

Supplemental Figure VI. Subcellular localisation of SMILR following siRNA induced knockdown. (A) qRT-PCR of NEAT1, a nuclear lncRNA, expression showing the clean separation of nuclear and cytoplasmic fractions of vSMCs, n = 3 biological replicates. (B) qRT-PCR showing the percentage changes in localisation of SMILR following siRNA induced knockdown. * = $p < 0.05$, n = 3 biological replicates. (C) qRT-PCR showing the relative quantification of SMILR knockdown in the nuclear and cytoplasmic fraction, * = $p < 0.05$, n = 3 biological replicates. All graphs via Iman and Conover non parametric analysis followed by students t test. (D) RNA-FISH showing SMILR (red) distribution in vSMCs stained with DAPI (blue) under siCtrl and siSMILR conditions using fluorescent microscopy at 40x magnification. Scale bars at 50 uM.

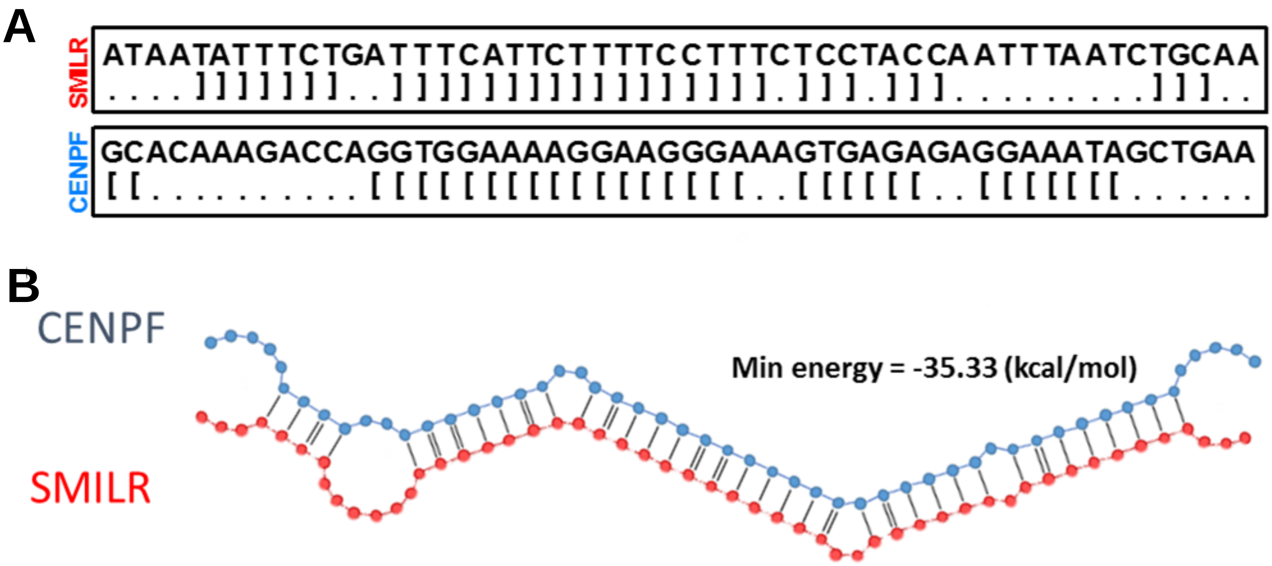

Supplemental Figure VII. Predicted interaction between SMILR and CENPF
(A) Predicted nucleotide interaction between SMILR and CENPF.
(B) Predicted secondary structure of SMILR:CENPF interaction.

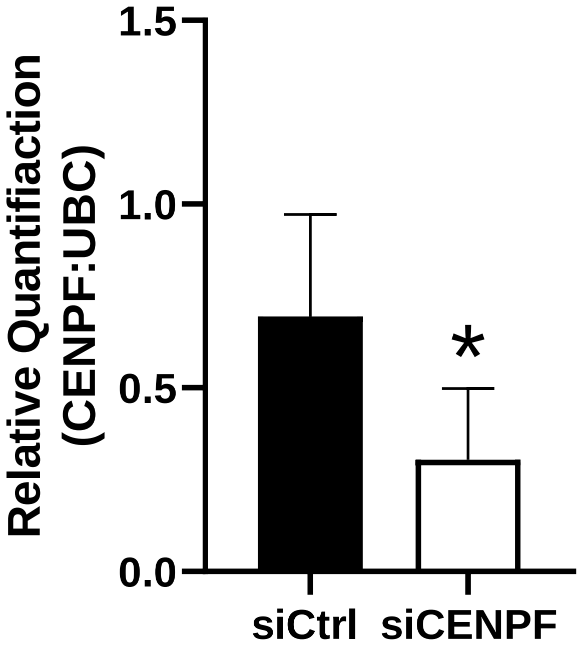

Supplemental Figure VIII: Confirmation of CENPF knockdown. qRT-PCR confirmating CENPF knockdown using dsiRNA (25nM). Data analysed via non parametric Iman and Conover ranked analysis followed by students t test * = $p < 0.05$ (n=4).

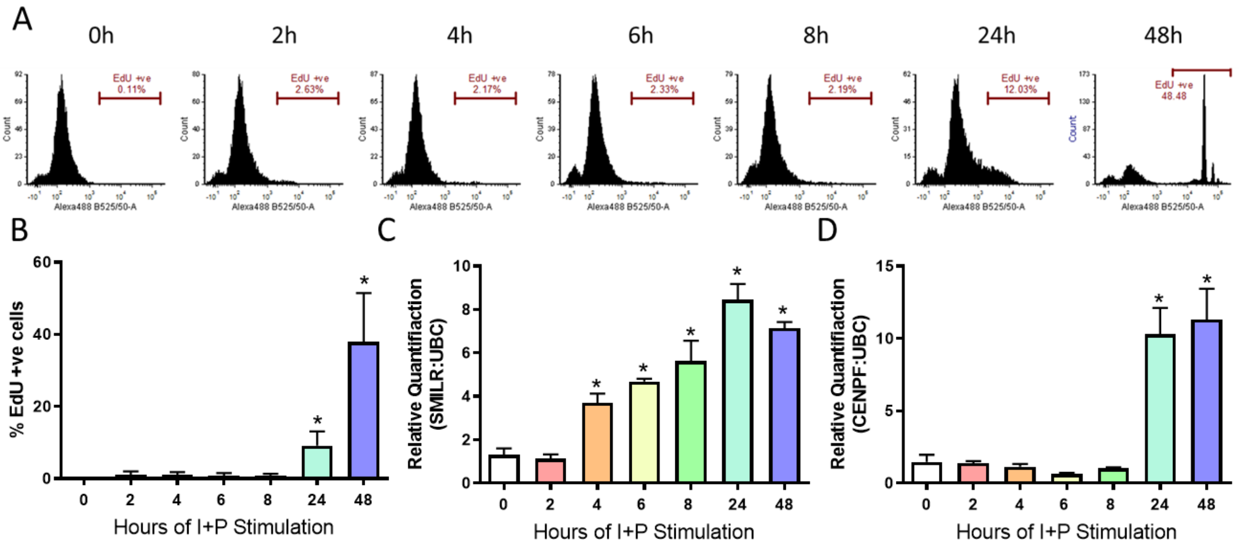

Supplemental Figure IX: Time course studies on IL1-PDGF stimulated HSVSMCs. (A) Representative FACs histogram plots showing % of EdU +ve cells at 0, 2, 4, 6, 8, 24, 48 hours post IL1-PDGF stimulation. (B) Bar chart representing the average of % EdU +ve cells with time, * = $p < 0.05$, n = 3 biological replicates. (C) Bar chart representing the average expression of SMILR by qRT-PCR with time, * = $p < 0.05$, n = 3 biological replicates. (D) Bar chart representing the average expression of CENPF by qRT-PCR with time, * = $p < 0.05$, n = 3 biological replicates. All graphs analysed via Iman and Conover ranked analysis followed by one way ANOVA.

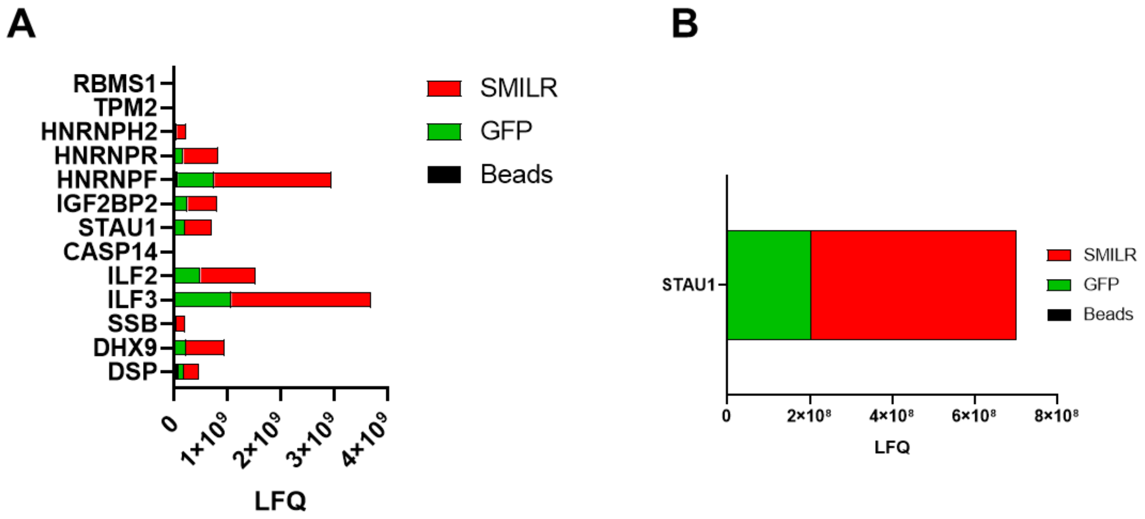

Supplemental Figure X: Biotinylated SMILR pulldown. A) Confirmation results of biotinylated SMILR pulldown with additional biotinylated GFP. Results from mass spectrometry analysis. LFQ (label free quantification) B) Levels of stau1 bound to beads, GFP or SMILR.

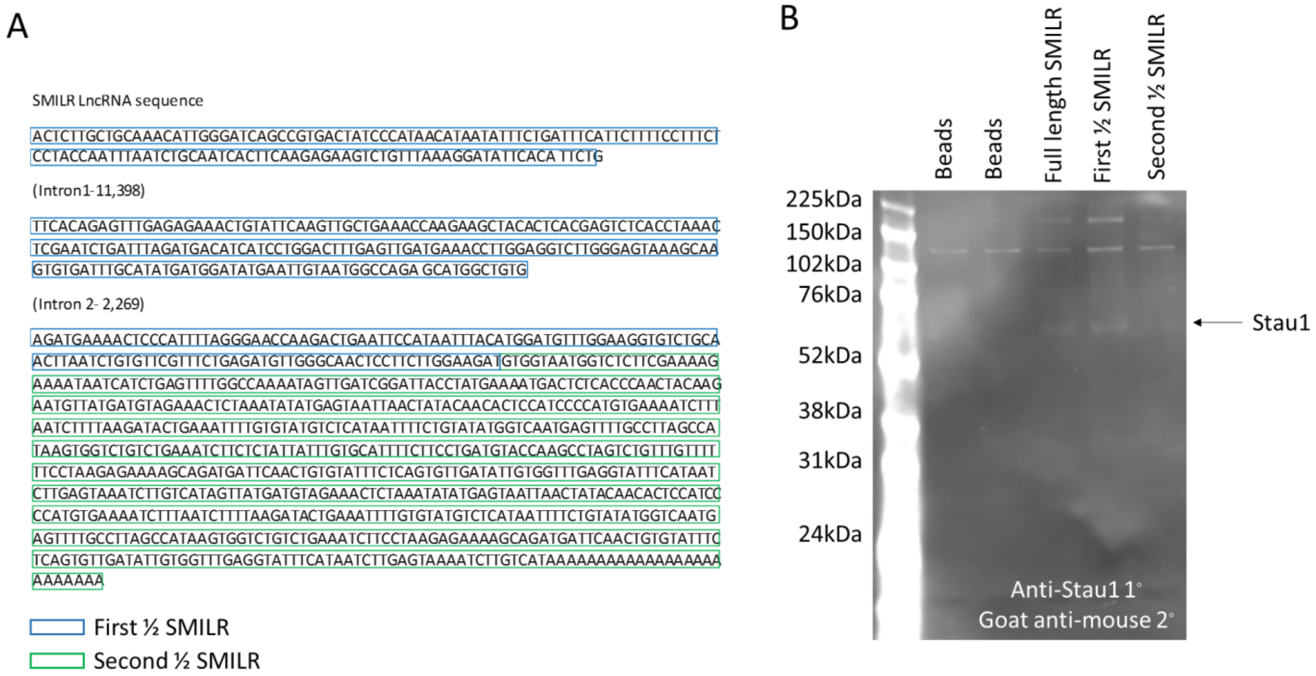

Supplemental Figure XI: Interaction of full length and truncated SMILR fragments with Stau1 protein. A) Schematic representation of the SMILR fragments used in this study. First ½ SMILR is denoted as blue and second ½ denoted as green boxes. Full length represents the full sequence of SMILR. B) Biotinylated constructs, denoted in a, or bead only controls were utilised as bait and incubated with protein lysates. Streptavidin beads were utilised to capture bound proteins and washed with wash buffer to remove non-specific interactions. Captured proteins were resolved by SDS-PAGE followed by immunoblotting with Stau1 primary antibody. Arrowhead represents expected size of Stau1 protein (55kDa).

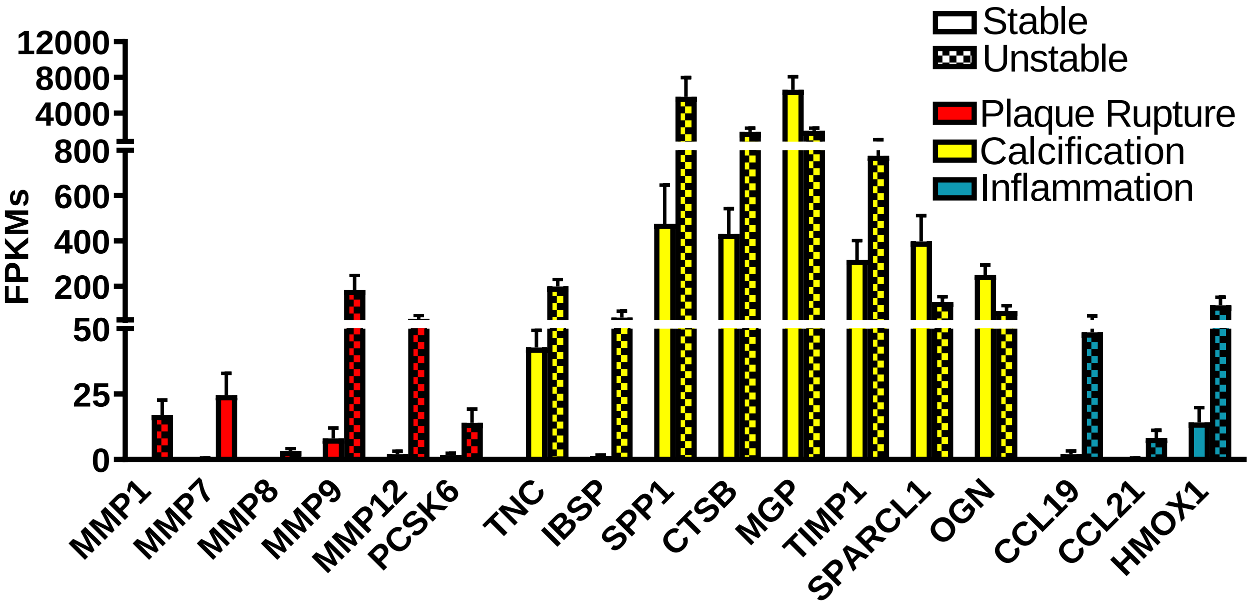

Supplemental Figure XII: Relative FPKMs of markers of plaque rupture, calcification, and inflammation in the stable versus unstable plaques.

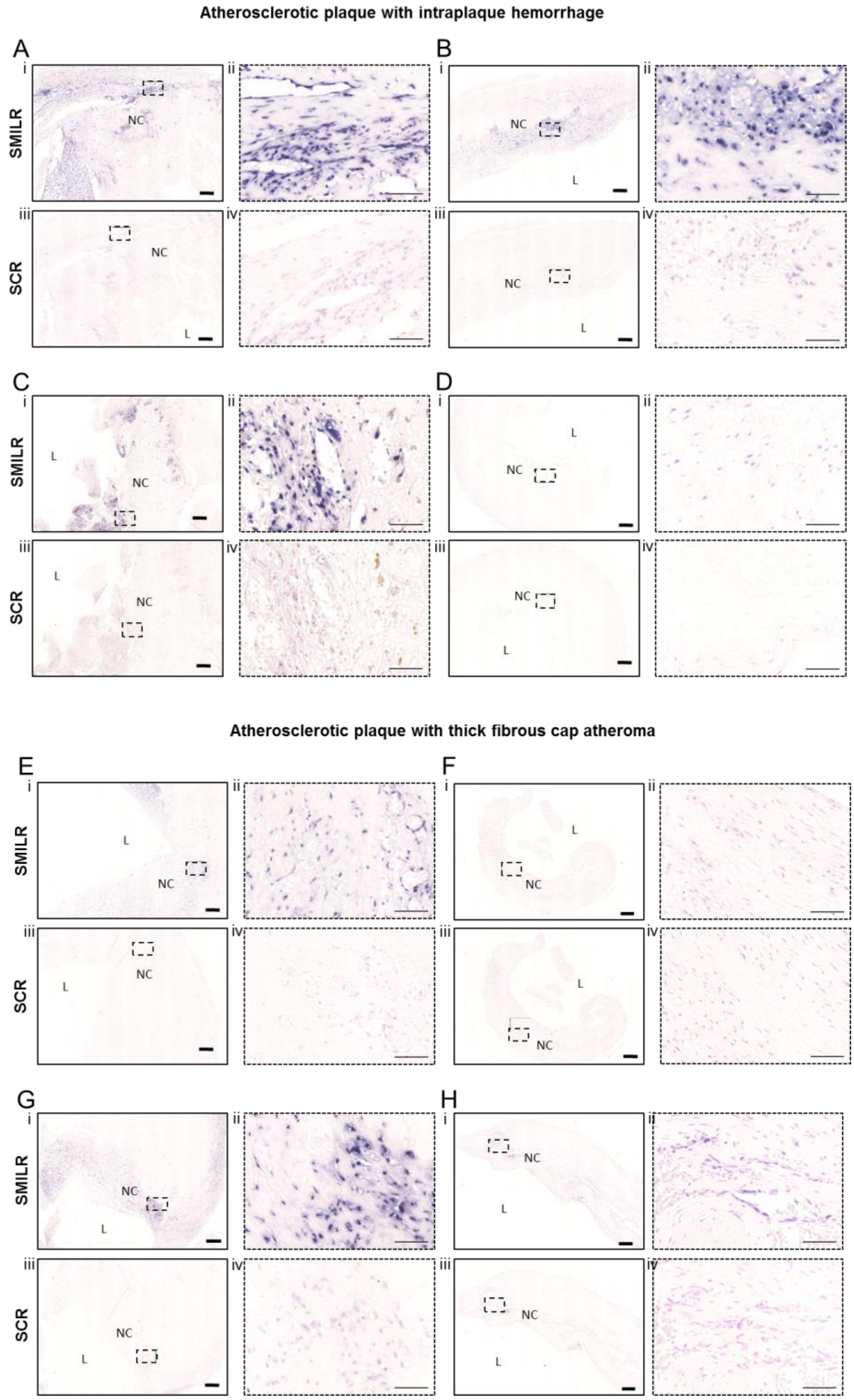

Supplemental Figure XIII. Localization of SMILR in human atherosclerotic plaques. Representative images of in situ detection of SMILR in 4 independent biological replicates of plaques in the carotid artery derived from symptomatic patients at carotid endarterectomy. SMILR is present in 4 of 5 plaques with intraplaque haemorrhaging using NBT/BCIP (purple), and present in 3 of 5 plaques with thick fibrous cap ahteroma in varying intensity (i, ii), while signal is absent after in situ hybridization with a scrambled (SCR) probe (iii, iv). Nuclei are stained with fast red. L, indicates arterial lumen; NC, lipid core; scale bar represents 200 μm.

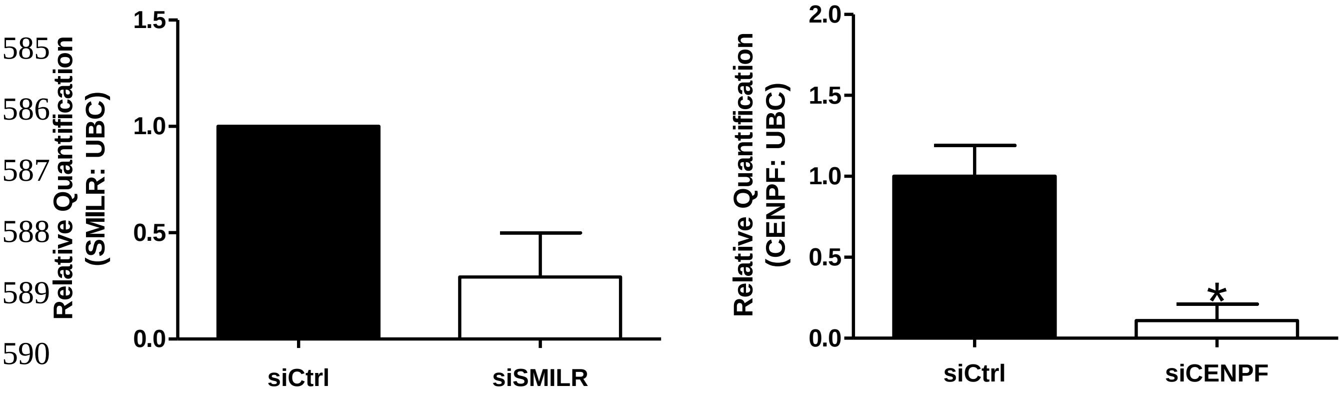

Supplemental Figure XIV. qRT-PCR confirming knockdown of (A) SMILR and (B) CENPF in CASMCs. Iman and Conover non parametric ranked analysis followed by students t test* = $p < 0.05$ by paired t-test. $n = 3$ biological replicates.

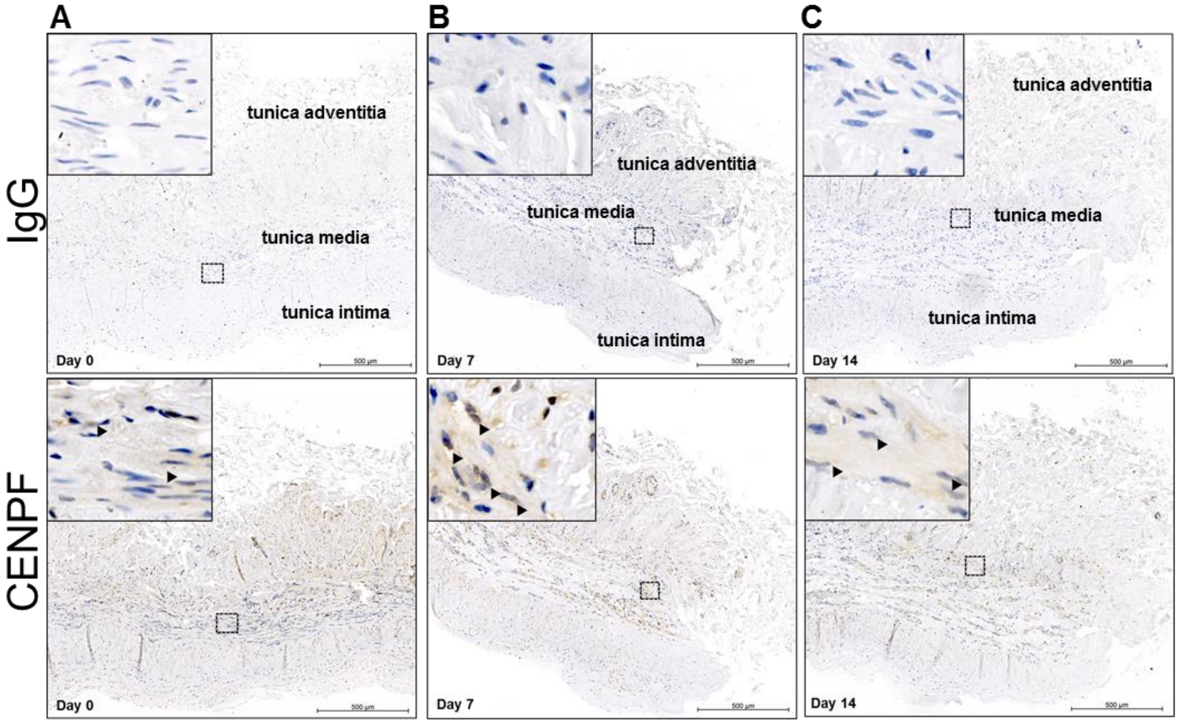

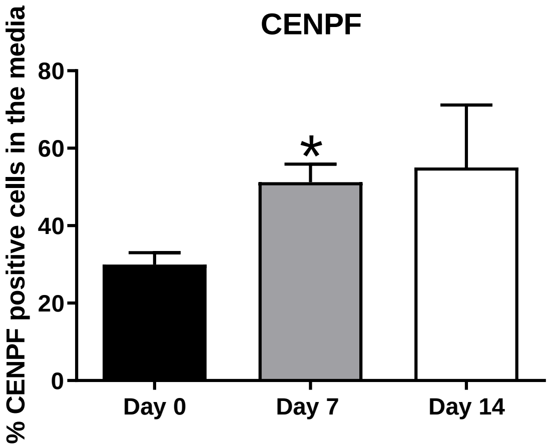

Supplemental Figure XV. CENPF protein levels are increased in the medial layer of cultured HSV. Representative images of medial CENPF protein levels at (A) day 0 and (B) day 7. Top panels corresponds to IgG negative control while bottom panels corresponds to CENPF; black arrows mark CENPF positive cells; scale bar is equal to 500 µm. (C) Quantification of medial CENPF IHC signal expressed as a percentage of CENPF positive cells. Iman and Conover non parametric ranked analysis * = $p < 0.05$.

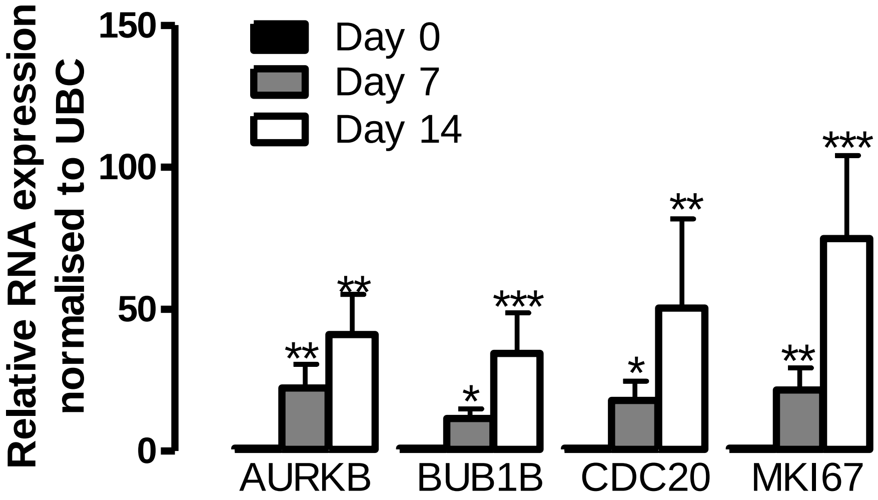

Supplemental Figure XVI: The relative quantification of (D) *SMILR*, (E) *CENPF*, and (F), other *SMILR* downstream targets within the cell cycle network, at day 0, 7 and 14. * = $p < 0.05$, Iman and Conover ranked non parametric analysis followed by students t test (n=3).

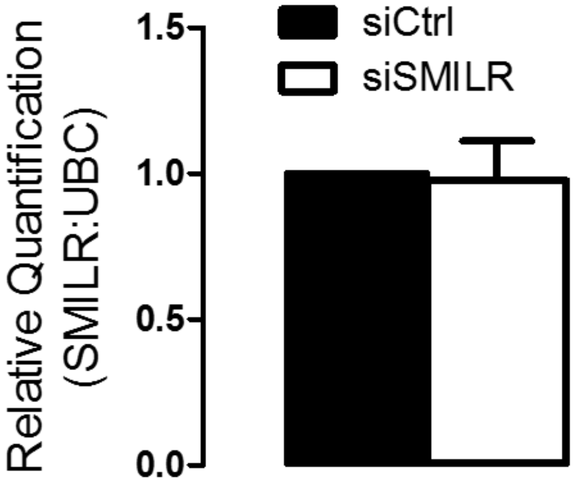

Supplementary Figure XVII. The loss of siRNA effects on SMILR knockdown at day 14 of intervention, n = 2 biological replicates**.**

Ranking	Gene	Average FPKM CTRL	LogFC SMILR overexp	FDR SMILR overexp	LogFC SMILR depletion	FDR SMILR depletion	logFC IL+PDGF	FDR IL+PDGF
1	TTN	0.13	-	-	-	-	-	-
2	KCNQ1OT1	0.65	-	-	-	-	-	-
3	MUC16	0.00	-	-	-	-	-	-
4	TSIX	0.04	-	-	-	-	-	-
5	FSIP2	2.00	0.40	5.64E-02	-0.35	6.11E-02	-	-
6	DST	84.46	0.11	7.19E-01	0.00	1.00E+00	-	-
7	NEB	0.21	-	-	-	-	-	-
8	GRIN2B	0.01	-	-	-	-	-	-
9	SYNE1	26.80	-0.02	9.40E-01	-0.01	9.46E-01	-	-
10	**SYNE2**	1.06	0.47	5.33E-02	-0.76	1.68E-08	-1.43482	0.005296
11	MACF1	82.14	0.12	3.82E-01	0.04	7.40E-01	-	-
12	SLC8A1	7.34	0.05	8.05E-01	-0.16	6.40E-02	-	-
13	DNAJC10	90.61	0.01	9.51E-01	0.14	2.00E-02	-	-
14	CNKSR3	1.78	0.09	8.03E-01	0.03	9.29E-01	-	-
15	RPAP2	7.15	0.13	3.06E-01	-0.25	2.66E-03	-	-
16	DNAH14	6.86	-0.06	8.96E-01	0.03	9.47E-01	-	-
17	XKR4	0.01	-	-	-	-	-	-
18	NCKAP1	31.49	0.12	1.41E-01	-0.38	1.64E-12	-	-
19	PCLO	0.08	-	-	-	-	-	-
20	AKAP9	7.80	0.11	4.35E-01	0.02	9.23E-01	-	-
21	CEP350	13.70	0.07	5.80E-01	-0.10	1.56E-01	-	-
22	ABI2	11.46	0.06	6.94E-01	0.08	3.59E-01	-	-
23	KIAA1109	11.83	0.07	7.26E-01	0.16	1.17E-02	-	-
24	SACS	15.60	0.22	1.47E-03	-0.17	6.34E-02	-	-
25	AGO3	6.82	0.18	7.86E-02	0.15	7.81E-02	-	-
26	XIRP2	0.02	-	-	-	-	-	-
27	ABCA13	0.15	-	-	-	-	-	-
28	BDP1	6.11	0.07	5.99E-01	0.02	9.19E-01	-	-
29	VPS13A	4.97	0.07	7.14E-01	-0.25	1.23E-03	-	-
30	GUCY1A2	0.75	-	-	-	-	-	-
31	CMYA5	0.07	-	-	-	-	-	-
32	RIF1	9.05	0.14	1.30E-01	0.07	5.66E-01	-	-
33	ZBTB37	3.17	0.14	3.94E-01	0.08	6.15E-01	-	-
34	DLX6-AS1	0.02	-	-	-	-	-	-
35	GPR98	other name					-	-
36	STX7	19.63	-0.03	8.52E-01	0.11	1.39E-01		
37	HMCN1	16.20	0.17	2.51E-01	0.42	1.82E-14		
38	LPP	9.38	0.22	2.26E-01	-0.57	4.01E-08		
39	DNAH8	0.00	-	-	-	-		
40	GOLGB1	15.91	-0.06	7.33E-01	0.07	3.09E-01		
41	**CENPF**	5.39	0.76	8.25E-25	-0.63	1.47E-31	3.258644	6.82E-06
42	KCNJ6	0.00	-	-	-	-		
43	**ANK3**	2.58	0.41	1.14E-02	0.94	2.17E-10	-1.01659	0.503041
44	DMD	4.73	-0.11	4.24E-01	-0.25	2.47E-03		
45	BRWD1	8.69	0.12	2.16E-01	0.02	9.12E-01		
46	FAT3	0.19	-	-	-	-		
47	**NEAT1**	13.91	-0.09	8.10E-01	-1.21	2.02E-10	-0.29834	0.852367
48	PPIP5K2	24.01	0.13	1.19E-01	0.08	2.48E-01		
49	**CENPE**	3.14	0.63	1.55E-10	-0.43	1.58E-05	2.317095	0.000171
50	KMT2C	6.02	0.15	1.33E-01	-0.05	5.74E-01		

51	DGKH	2.44	0.15	3.00E-01	0.08	6.90E-01		
52	XIST	0.03	-	-	-	-		
53	DYNC2H1	8.28	0.17	4.51E-02	-0.16	1.28E-01		
54	ATRX	22.59	0.06	6.35E-01	0.06	4.58E-01		
55	BOD1L1	7.29	0.07	6.17E-01	0.07	3.65E-01		
56	RYR2	0.00	-	-	-	-		
57	USH2A	0.01	-	-	-	-		
58	DNAH5	0.75	-	-	-	-		
59	HOOK3	8.90	0.06	6.55E-01	-0.18	1.02E-02		
60	DNAH11	1.11	-0.27	6.69E-01	-0.17	7.63E-01		
61	TNRC6B	5.00	0.16	1.10E-01	-0.04	7.62E-01		
62	ANKRD12	11.84	-0.02	9.54E-01	-0.08	4.38E-01		
63	ONECUT2	0.00	-	-	-	-		
64	AHNAK	76.66	0.16	3.57E-01	0.15	8.88E-02		
65	ITSN1	14.41	0.11	2.18E-01	0.27	7.23E-07		
66	FZD3	0.41	-	-	-	-		
67	MDN1	3.97	0.07	5.41E-01	-0.05	5.21E-01		
68	ANK2	20.94	0.03	8.14E-01	0.39	3.25E-11		
69	LRP1B	0.01	-	-	-	-		
70	ZNF704	0.23	-	-	-	-		
71	C5orf42	4.56	0.13	2.97E-01	0.04	7.68E-01		
72	CEP290	7.95	0.05	7.76E-01	-0.02	8.97E-01		
73	VPS13C	14.22	-0.04	7.65E-01	0.12	1.08E-01		
74	KMT2A	4.53	0.17	8.48E-02	0.02	9.08E-01		
75	NFAT5	3.63	0.06	8.76E-01	-0.05	7.12E-01		
76	POU2F1	1.09	-0.03	9.48E-01	-0.01	9.89E-01		
77	RNF213	14.07	-0.18	5.24E-01	0.06	5.10E-01		
78	PKHD1	0.00	-	-	-	-		
79	MYCBP2	19.30	0.05	8.68E-01	0.07	4.19E-01		
80	FAT4	1.63	0.08	7.08E-01	-0.09	3.74E-01		
81	DGKI	1.74	0.00	1.00E+00	-0.40	6.03E-04		
82	UBN2	1.39	0.22	1.86E-01	-0.13	4.36E-01		
83	ATM	23.18	0.09	4.48E-01	0.09	2.52E-01		
84	SUGT1	39.64	0.06	6.42E-01	-0.07	3.07E-01		
85	BIRC6	21.60	0.03	8.03E-01	-0.04	7.17E-01		
86	GOLGA4	24.70	0.10	2.40E-01	-0.05	5.93E-01		
87	APOB	0.00	-	-	-	-		
88	LYST	7.70	-0.06	6.20E-01	-0.46	3.24E-16		
89	BRCA2	2.32	0.27	1.27E-02	-0.11	6.03E-01		
90	KCTD16	0.75	-	-	-	-		
91	APC	13.51	0.14	3.46E-01	0.24	4.91E-05		
92	RANBP2	17.82	0.06	6.20E-01	0.06	4.59E-01		
93	CHD9	13.46	-0.03	8.31E-01	0.12	1.01E-01		
94	CAMK4	0.34	-	-	-	-		
95	MMP16	7.61	0.25	6.00E-03	0.31	1.53E-03		
96	HERC1	13.48	0.09	3.34E-01	0.05	5.68E-01		
97	INO80D	1.73	0.14	3.04E-01	-0.15	5.96E-01		
98	PDK1	5.29	-0.04	8.97E-01	0.22	2.68E-02		
99	CCDC88A	10.34	0.18	1.15E-02	0.07	4.03E-01		
100	**ASPM**	3.88	0.81	1.73E-30	-0.67	4.57E-14	0.784365	0.489931

Supplemental Table I: Ranking based on SumEnergy of the top100 predicted SMILR-interacting RNAs.

	Forward (5'-3')	Reverse (5'-3')
T7 SMILR	GATACCTAATACGACTCACTATAGGGGATACCCACCATGGACTCTTGCTGCAAACAT	TTTTTTTTTTTTTTTTTTTTTTTTTTTTTTTTAATGACAAGATTTTACTCAAGAT
T7 GFP	GATACCTAATACGACTCACTATAGGGGATACCCACCATGGATGGTGAGCAAGGGCGA	TTTTTTTTTTTTTTTTTTTTTTTTTTTTTTTTACTTGTACAGCTCGTCCATGC
SMILR	ACCTTGGAGGTCTTGGGAGT	TTGCAGACACCTTCCAAACA
UBC	TTGCCTTGACATTCTCGATG	ATCGCTGTGATCGTCACTTG
AMA1	CAGTGGCTCATGTCGAGTTTCCAGA	CGACCTTTCTTTCCATCATTCATCGG

Supplemental Table II: T7 primers for *in vitro* transcription and SYBR primers for qRT-PCR

dsiRNAs	Sequence (5' – 3')	Concentration (nM)
Control	CUUCCUCUCUUUCUCUCC	10/25
SMILR	CCAUAAUUUACAUGGAUG	25
STAU1	GUCAAGGACAUCACACCA	10
CENPF	GAAUGAUUCACUUAAGGA	25
AURKB	GGUGAUUCACAGAGACAUAAAGCCA	25

Supplemental Table III: IDT Designed dsiRNAs and concentrations used.

**Taqman Probe**	**Assay Number**
UBC	Hs01871556_s1
STAU1	Hs00244999_m1
AURKB	Hs00945858_g1
BUB1B	Hs01084828_m1
AMA1	Ce02462726_m1
CCNA2	Hs00996788_m1
CCNE2	Hs00180319_m1
CDC20	Hs00426680_mH
CENPA	Hs00156455_m1
CENPF	Hs01118845_m1
KIF15	Hs01085295_m1
KIF20A	Hs00993573_m1
MKI67	Hs01032443_m1
NCAPG	Hs00254617_m1
CLSPN	Hs00898637_m1
ESCO2	Hs00411577_m1
EXO1	Hs01116190_m1
MYBL2	Hs00942540_m1
NUSAP1	Hs01006195_m1
PBK	Hs00902990_m1
SKA1	Hs00536843_m1
SPC24	Hs00699347_m1
UBE2C	Hs00964100_g1
XRCC2	Hs03044154_m1

Supplemental Table IV: Taqman Probes for qRT-PCR

Antisense Probes	Sequence (5’-3’)
GFP Odd 1	aaatttgtgcccattcacatcgccatccagttccacgaga/3BioTEG/
GFP Odd 2	aaagcactgcacgccataagagaaggtagtgaccagtgtt/3BioTEG/
GFP Odd 3	agcgcgggtcttgtagttcccgtcatctttgaaaaagatg/3BioTEG/
GFP Odd 4	gagttatagttgtattccagcttgtggccgagaatgtttc/3BioTEG/
GFP Odd 5	gctgcacggatccatcctcaatgttgtgtctgatcttgaa/3BioTEG/
SMILR Odd 1	atgttatgggatagtcacggctgatcccaatgtttgcagc/3BioTEG/
SMILR Odd 2	gattcgagtttaggtgagactcgtgagtgtagcttcttgg/3BioTEG/
SMILR Odd 3	tccatgtaaattatggaattcagtcttggttccctaaaat/3BioTEG/
SMILR Odd 4	ataggtaatccgatcaactattttggccaaaactcagatg/3BioTEG/
SMILR Odd 5	aaagattttcacatggggatggagtgttgtatagttaatt/3BioTEG/
SMILR Even 1	gtgaatatcctttaaacagacttctcttgaagtgattgca/3BioTEG/
SMILR Even 2	ccattacaattcatatccatcatatgcaaatcacacttgc/3BioTEG/
SMILR Even 3	gaaggagttgcccaacatctcagaaacgaacacagattaa/3BioTEG/
SMILR Even 4	tacatcataacattcttgtagttgggtgagagtcattttc/3BioTEG/
SMILR Even 5	atttcagacagaccacttatggctaaggcaaaactcattg/3BioTEG/

Supplemental Table V: Antisense 3’end biotinylated DNA probes for *SMILR* and *GFP* RNA:RNA pulldowns
